# Production and Characterization of ACE Inhibitory and Anti-Diabetic Peptides from Buffalo and Camel Milk Fermented with Lactobacillus and Yeast: A Comparative Analysis with In Vitro, In Silico, and Molecular Interaction Study

**DOI:** 10.3390/foods12102006

**Published:** 2023-05-15

**Authors:** Ruchita Khakhariya, Bethsheba Basaiawmoit, Amar A. Sakure, Ruchika Maurya, Mahendra Bishnoi, Kanthi Kiran Kondepudi, Srichandan Padhi, Amit Kumar Rai, Zhenbin Liu, Subrota Hati

**Affiliations:** 1 Department of Dairy Microbiology, SMC College of Dairy Science, Kamdhenu University, Anand 388110, Gujarat, India; k.ruchita11@gmail.com; 2 Department of Rural Development and Agricultural Production, Tura Campus, North-Eastern Hill University, Chasingre 794002, Meghalaya, India; bethsheba111@gmail.com; 3 Department of Agriculture Biotechnology, Anand Agricultural University, Anand 388110, Gujarat, India; sakure.amar455@gmail.com; 4 Regional Center for Biotechnology, Faridabad 121001, Haryana, India; ruchika6566maurya@gmail.com; 5 Healthy Gut Research Group, Food & Nutritional Biotechnology Division, National Agri-Food Biotechnology Institute, Knowledge City, Sector 81, SAS Nagar 140306, Punjab, India; mbishnoi@nabi.res.in (M.B.); kiran@nabi.res.in (K.K.K.); 6 Institute of Bioresources and Sustainable Development, Regional Centre, Tadong 737102, Sikkim, India; srichandan1989@gmail.com (S.P.); amitrai.ibsd@gov.in (A.K.R.); 7 School of Food and Biological Engineering, Shaanxi University of Science and Technology, Xi’an 710021, China; lzbrcy@126.com; 8 Department of Dairy Microbiology, Kamdhenu University, Anand 388110, Gujarat, India

**Keywords:** anti-hypertensive, anti-diabetic, anti-inflammatory activity, FTIR, RP-HPLC, RPLC/MS

## Abstract

The investigation aimed at assessing a comparative study on the production and characterization of ACE inhibitory, anti-diabetic, and anti-inflammatory activities, along with the production of ACE inhibitory and anti-diabetic peptides through the fermentation of buffalo and camel milk by *Limosilactobacillus fermentum* (KGL4) and *Saccharomyces cerevisiae* (WBS2A). The angiotensin-converting enzyme (ACE) inhibitory and anti-diabetic properties were evaluated at particular time intervals (12, 24, 36, and 48 h) at 37 °C, and we discovered maximum activity at 37 °C after 48 h of incubation. The maximum ACE inhibitory, lipase inhibitory activities, alpha-glucosidase inhibitory, and alpha-amylase inhibitory activities were found in the fermented camel milk (77.96 ± 2.61, 73.85 ± 1.19, 85.37 ± 2.15, and 70.86 ± 1.02), as compared to the fermented buffalo milk (FBM) (75.25 ± 1.72, 61.79 ± 2.14, 80.09 ± 0.51, and 67.29 ± 1.75). Proteolytic activity was measured with different inoculation rates (1.5%, 2.0%, and 2.5%) and incubation times (12, 24, 36, and 48 h) to optimize the growth conditions. Maximum proteolysis was found at a 2.5% inoculation rate and at a 48 h incubation period in both fermented buffalo (9.14 ± 0.06) and camel milk (9.10 ± 0.17). SDS-PAGE and 2D gel electrophoresis were conducted for protein purification. The camel and buffalo milk that had not been fermented revealed protein bands ranging from 10 to 100 kDa and 10 to 75 kDa, respectively, whereas all the fermented samples showed bands ranging from 10 to 75 kDa. There were no visible protein bands in the permeates on SDS-PAGE. When fermented buffalo and camel milk were electrophoresed in 2D gel, 15 and 20 protein spots were detected, respectively. The protein spots in the 2D gel electrophoresis ranged in size from 20 to 75 kDa. To distinguish between different peptide fractions, water-soluble extract (WSE) fractions of ultrafiltration (3 and 10 kDa retentate and permeate) of fermented camel and buffalo milk were employed in RP-HPLC (reversed-phase high-performance liquid chromatography). The impact of fermented buffalo and camel milk on inflammation induced by LPS (lipopolysaccharide) was also investigated in the RAW 264.7 cell line. Novel peptide sequences with ACE inhibitory and anti-diabetic properties were also analyzed on the anti-hypertensive database (AHTDB) and bioactive peptide (BIOPEP) database. We found the sequences SCQAQPTTMTR, EMPFPK, TTMPLW, HPHPHLSFMAIPPK, FFNDKIAK, ALPMHIR, IPAVFK, LDQWLCEK, and AVPYPQR from the fermented buffalo milk and the sequences TDVMPQWW, EKTFLLYSCPHR, SSHPYLEQLY, IDSGLYLGSNYITAIR, and FDEFLSQSCAPGSDPR from the fermented camel milk.

## 1. Introduction

Milk is known as a near-complete food due to the presence of functional and nutritious components [1]. Milk is an essential food in the diet of the majority of the world’s residents, and due to its significant nutritional benefits, it is often recognized as a complete diet. Protein, fat, carbohydrates, vitamins, and minerals are all present in sufficient amounts in milk. As a result of milk’s nutritional significance, newly born mammals rely on it as a primary source of nourishment until they can digest other foods [2].

The nutrients in milk are crucial for maintaining adult health. Bovine milk has a pH of 6.6, and the average parameters of milk constituents are 87.2% water, 4.9% lactose, 3.7% fat, 0.7% ash, and 3.5% protein. Milk constituents differ according to the animal’s stage of lactation, age, breed, animal nutrition, and health status [3]. Buffalo milk contains 6.7% fat, 4.7% protein, 4.7% lactose, 0.8% ash, 16.3% total solids, and 83.7% water [4], whereas camel milk has 87% water, 11.9% total solids, 4.4% lactose, 3.1% protein, 0.79% ash, 3.5% fat, and 4.4% lactose [5]. A number of health advantages are provided by milk-borne bioactive peptides (BAPs), including anti-carcinogenic, anti-hypertensive, anti-oxidant, anti-pyretic, immunomodulatory, anti-microbial, and energy-burning capabilities as well as appetite regulation [6].

According to Yasmin et al. [7], sheep milk has the highest crude protein (CP) level (4.35%), followed by buffalo milk (4.17%), and cow and camel milk have the lowest (3.56% and 3.38%, respectively). Huma et al. [8] discovered that the total proteins in buffalo, cow, sheep, and camel milk were equivalent, while goat milk had an even higher protein level. Cow, goat, and sheep milk had protein contents of 3.4%, 3.7%, and 5.5%, respectively [9]. The protein level of milk has a significant impact on its nutritional and technological value. Major protein fractions, such as CP, true proteins (TP), caseins, and whey proteins, as well as nitrogen components, such as non-casein nitrogen (NCN) and non-protein nitrogen (NPN) concentrations, differed amongst the milk types. Sheep milk had the highest levels of caseins, CP, TP, and NPN, while camel milk had the lowest. The variability in protein content between different animal species is related to genetic diversity. Whey protein content was the highest in sheep milk (0.74%), whereas it was the lowest in cow milk (0.53%). The NCN content in sheep milk was higher (1.31%), while it was lower in camel (0.91%) and goat milk (0.90%). However, sheep milk had the highest NPN level (0.57%), followed by buffalo milk (0.52%). [7]. Sheep milk was high in casein (4.2–5.2 g/100 g) and whey proteins (1.02–1.3 g/100 g) [10,11], but camel milk had a protein content ranging from 2.15 to 4.90% [12].

Buffalo milk, which is widely consumed around the world, is a relatively underexplored source of BAPs. Hamid et al. [13] investigated the ACE inhibitory and anti-oxidative activities of buffalo milk peptides. Additionally, camel milk is also described as a high-protein source with an ACE inhibitory effect [14]. Tagliazucchi et al. [15] investigated the anti-hypertensive activity of gastrointestinal digested camel milk hydrolysate (in vitro). In protein hydrolysates produced from camel milk, Mudgil et al. [16] found different anti-diabetic and anti-obesity peptides (KDLWDDFKGL and MPSKPPLL).

Fermentation is an ancient food preservation technique where bioactive peptides can be formed. Lactic acid bacteria (LAB) are generally fermented naturally or under controlled conditions to make fermented foods. Due to their ability to contribute nutritional and technological qualities, such as texture and flavor development, LAB are essential in the invention of fermented foods [17]. In order to functionalize milk byproducts and products, the dairy industry has already used the economically advantageous method of microbial fermentation to yield BAPs [18,19]. Therefore, Lactobacillus species fermentation can result in the improvement of foods that are healthy, allowing simultaneous development and functionalization. During fermentation, Lactobacillus species produce a wide range of metabolites, including lactate, vitamins, or exopolysaccharides, in addition to bioactive peptides. These can all have a positive impact on human health [20].

In milk fermentation processing, yeast has been developed as a natural fermentation strain as a result of continued research on starter cultures, and it is the second most important dairy microflora after LAB. Previously, it was believed that yeast might result in spoiling, undesirable odors, and abnormalities; however, recent research indicates that particular yeasts can be employed as secondary starters during fermentation and maturation to alter the structure of milk products [21]. The flavor, texture, value, and structure of milk products are influenced by the yeasts’ abilities to create aroma precursors and hydrolyze proteins. Yeast may break down lactose and galactose and produce flavorings such as ethanol and glycerol to give fermented milk a tasty flavor when employed as a secondary starter culture [22]. In addition to providing potential prebiotics for human consumption, the simultaneous generation of alcohol and CO_2_ (carbon dioxide) can stop the development of harmful microorganisms [23]. Milk protein hydrolysates are often produced through fermentation and depend on the proteolytic path of the LAB added; this has been described as an effective and secure method for producing food-grade hydrolysates and BAPs [19]. 

In this investigation, the anti-diabetic and anti-hypertensive properties of fermented buffalo (*Bubalus bubalis*) and camel (*Camelus dromedaries*) milk were examined. The ability of fermented buffalo and camel milk to reduce inflammation in the RAW 264.7 macrophage cell line was also examined. The goal of this study was to see how well novel peptides found in fermented buffalo and camel milk worked as ACE inhibitors and anti-diabetics.

## 2. Materials and Methods

### 2.1. Collection of Bacterial Cultures

*Limosilactobacillus fermentum* (KGL4, MF951099) was obtained from the Culture Collection of the Dairy Microbiology Department at Kamdhenu University, Anand, Gujarat, India, and *Saccharomyces cerevisiae* (WBS2A, MG101828) yeast culture was obtained from the R.D.A.P. department at North-Eastern Hill University, Tura, Meghalaya, India.

### 2.2. Sample Preparation

To maintain 11% TS, camel milk powder obtained from GCMMF Ltd., Anand, India, was diluted in water; a 10 min, 90 °C heat treatment was administered, and the mixture was then stored at 5 °C for future research. From a typical commercial provider (GCMMF Ltd., Anand, India), buffalo milk was purchased, followed by 15 min of sterilization at 121 °C under 15 psi, followed by storage at 5 ± 1 °C. Two percent inoculum was added to the buffalo milk and camel milk, respectively, to activate the pure cultures; the LAB cultures were incubated at 37 °C for 24 h, and the yeast cultures were incubated at 25 °C for 3–5 days. In addition, samples from the heat-treated buffalo and camel milk were examined after 2% inoculation with Lactobacilli and yeast cultures. These samples were then maintained for various incubation times at 37 °C (12, 24, 36, and 48 h). Following incubation for 12, 24, 36, and 48 h, the fermented milk samples were centrifuged for 20 min at 4 °C at 5000 rpm. A 0.22 µm syringe filter was used to strain the supernatants after they were collected in order to ascertain their anti-diabetic and anti-hypertensive properties. For all the activities, three replications were carried out.

### 2.3. Evaluation of Camel and Buffalo Milk’s ACE Inhibitory Activity

To assess ACE inhibitory activity, 200 μL of sample supernatant, 200 μL of a 5 mM HHL substrate solution, and 0.5 mL of water (HPLC grade) were mixed. The method began with the addition of 20 μL of the ACE enzyme (4 mU in 250 μL), which was allowed to stand for 30 min at 37 °C. One thousand microliters of 1 N chilled hydrochloric acid was added to end the process. It was then incubated for another 30 min at 37 °C. Centrifugation at 5000 rpm (revolutions per minute) for 10 min extracted the hippuric acid produced by the ACE with 1.7 mL of ethyl acetate. After centrifugation, two layers formed, and the upper layer containing the hippuric acid was removed and evaporated at 100 °C for 10 min. After dissolving the hippuric acid-containing residue in 2 mL of deionized water, the absorbance of the sample at 228 nm was determined in comparison to a blank using the Indian-made Systronic Double Beam Spectrophotometer 2202. As a control, the unfermented sample was kept [24].

ACE inhibition (%) = [(A_control_ − A_ample_)/A_control_] × 100

where A_sample_ is the absorbance of the sample, and A_control_ is the absorbance of the control.

### 2.4. Evaluation of the Anti-Diabetic Properties of Fermented Camel and Buffalo Milk

#### 2.4.1. ɑ-Glucosidase Inhibition Efficacy

The mixture contained supernatant (100 μL) from the fermented camel and buffalo milk, 2.5 mL (1 U/mL) of the enzyme (ɑ-glucosidase), 1500 μL of phosphate buffer with 6.8 pH (100 mM), and 0.1 mL of phosphate buffer, incubated for 10 min at 37 °C. The mixture was then incubated at 37 °C for approximately 20 min with 0.5 mL of the substrate, 4-Nitrophenyl-D-glucopyranoside (P-NPG). The reaction was stopped by adding 100 μL of 0.1 M Na_2_CO_3_. The method employed by Shai et al. [25] and Yamaki and Mori [26] to determine free p-nitrophenols at 405 nm was used. The amount of inhibition obtained was calculated as follows:

ɑ-glucosidase inhibition (%) = [(A_control_ − A_sample_)/A_Control_] × 100

where A_sample_ is the sample absorbance, and A_control_ is the control sample absorbance.

#### 2.4.2. ɑ-Amylase Inhibition Activity

Preincubation was performed for five minutes at 37 °C with 20 μL of the enzyme (α-amylase), 0.4 mL of phosphate buffer with 6.8 pH (100 mM), and 200 μL of the supernatant from the fermented camel and buffalo milk. The substrate was then added, along with 0.2 mL of soluble starch (1%). Following a 20 min incubation period at 37 °C, 2 mL of the 3, 5-dinitrosalicylic acid (DNSA) colorant was added, and the mixture was then brought to a boil for 10 min. The absorbance was assessed at 540 nanometers. [27,28]. The percentage inhibition obtained was calculated using the process below:

ɑ-amylase inhibition (%) = [(A_control_ − A_sample_)/A_Control_] × 100

where A_sample_ is the absorbance of the sample, and A_control_ is the absorbance of the control sample.

#### 2.4.3. Lipase Inhibitory Activity

The pancreatic lipase inhibitory activity was determined by isolating the substrate 4-methylumbelliferyl oleate (4MUO) from 4-methylumbelliferone (4MU), in accordance with Kurihara et al. [29] and Sergent et al. [30]. The mixture was incubated at 37 °C for 30 min with 0.1 mL of supernatants from fermented camel and buffalo milk; 1700 μL of phosphate buffer with 6.8 pH (100 mM); 2 μL of an enzyme (pancreatic lipase) (0.5 mg/mL); and 0.1 mL of 4MUO (0.25 mM). The procedure was completed by adding 0.1 mL of sodium citrate (0.1 M). At 260 nm, the release of 4MU was observed. The following percentage of pancreatic lipase inhibitory action was calculated:

Lipase inhibition (%) = [(A_control_ − A_sample_)/A_Control_] × 100

where A_sample_ is the absorbance of the sample, and A_control_ is the absorbance of the control sample.

#### 2.4.4. Estimation of Proteolytic Activity

To determine the degree of proteolysis, the O-phthalaldehyde technique was used [24,31]. In order to maximize the generation of peptides in heat-treated camel milk and sterilized buffalo milk, several inoculum rates (1.5, 2.0, and 2.5%) and incubation times (12, 24, 36, and 48 h) were used. After each interval, samples were taken in order to determine the peptide content. Three milliliters of each sample was added to five milliliters of trichloroacetic acid (TCA), which was vortexed for one minute before being filtered using Whatman No. 42 filter paper (UK). After the filtrate (0.4 mL) and OPA reagent (3 mL) were combined and incubated at room temperature (20 °C) for 2 min, absorbance was then measured at 340 nm.

### 2.5. Sodium Dodecyl Polyacrylamide Gel Electrophoresis (SDS-PAGE) Analysis

To estimate the molecular weight of different protein fragments, SDS-PAGE was used. In the current investigation, a 12% separating gel was used for SDS-PAGE, and the test samples included WSEs of the fermented buffalo and camel milk [32,33,34].

### 2.6. 2D Gel Electrophoresis

In accordance with Yang et al. [35], the peptides produced by fermenting buffalo and camel milk were detected using 2D gel electrophoresis with minor changes to the electric parameters.

### 2.7. Isoelectric Focusing (IEF)

The test sample was prepared with 1000 µL of WSEs from the fermented camel and buffalo milk and cleaned with the Ready Prep 2D Clean-Up Kit (BioRad, Cat. No. 163-2130). A protein sample was applied to a 7 cm ready IPG strip at a concentration of approximately 125 g/mL (pH 3–10) (Bio-Rad). In accordance with Panchal et al. [36], the electrical thresholds were utilized for IEF. The strip was immersed in a solution once electrophoresis was finished. Afterwards, the strip was made ready for 2D gel electrophoresis analysis by soaking it for ten minutes in equilibrium buffer I (EB-I), ten minutes in equilibrium buffer II, and then two minutes in 1X Tris-Glycine Sodium Dodecyl Sulphate Buffer (Bio-Rad). Following SDS-PAGE, in-gel trypsin digestion was conducted using the Trypsin Profile IGD Kit.

### 2.8. Reversed-Phase High-Performance Liquid Chromatography for Peptide Separation

The development of peptides in the fermented buffalo and camel milk was measured, and the distinct peptide peaks were separated using a reversed-phase high-performance liquid chromatography system (Shimadzu LC-20, Kyoto, Japan). The RPHP-LC system with a binary gradient and a white pore analytic column measuring 5 µ, 250 × 4.6 mm from Thermo Fisher Scientific was employed. A micro-injector with a 20 μL loop from Hamilton Bonaduz AG, Switzerland, was used to inject the sample. Both eluent-A and eluent-B included TFA at concentrations of 0.01% (*v*/*v*) in deionized water and 0.01% (*v*/*v*) in an acetonitrile/deionized water combination, respectively (80:20). At room temperature, a 0.25 milliliter per minute flow rate was used for the segregation process. Elution timings for the peptides were followed in accordance with Parmar [37]. Using a detector (Shimadzu, SPD-20A) set up for both visible and UV wavelengths, a peak at 214 nm was discovered [37].

### 2.9. Identification and Characterization of Isolated Peptides by RPLC/MS

In accordance with Parmar [37], UPLC Ekspert UltraLC 100 (Eksigent) equipment was used in conjunction with QTRAP 4500 mass spectrometry. The column oven, degasser, automated autosampler, and binary pump system are all included in the UPLC. The peptides were eluted at 0.3 mL/min using a gradient program and an Adhoc C18-based column (2.1 mm × 150 mm) with a particle size of 1.5 m. The gradient elution procedure was precisely adjusted to allow for the UPLC’s maximum pressure of 8000 psi. The mobile phase included 0.1% formic acid, and the peptide was eluted in water and acetonitrile. For the electron spray ionization (ESI) interface, we employed an AB SCIEX QTRAP 4500 and an Ekspert Ultra-LC 100 from Eksigent, USA. RPLC/MS was utilized to discover unknown peptides and identify peptide sequences by using enhanced mass spectra and enhanced product ion scans. EMS was selected to obtain masses ranging from 350 to 2000 Da. The scan rate for the data acquisition was set to 10,000 Da, and the Linear Ion Trap’s Qo trapping mode permitted mass detection lasting 40 msec (LIT). Additionally, an enhanced mass resolution scan of EMS was performed at 250 Da with a 1 to 2 Da center isotope detection window. Additionally, information-dependent acquisition (IDA) was put into automatic optimization mode with rolling circle energy to choose the appropriate collision energy (CE). With a mass difference of 250 Da, the old target ion was preserved within the detection range. To reduce background and extraneous masses, the mass detector was allowed to perform background subtraction and identify masses at the above intensity of 5000 cps. With a scan rate of 10,000 Da/sec, EPI was then used to fragment the former target ion between 50 and 1000 Da. To improve the product mass spectra, 40 msec of LIT was utilized in conjunction with the Q0 trapping function.

### 2.10. Data Analysis and Identification of Peptides

The ACE inhibitory activity of the peptides was verified using the anti-hypertensive peptide database (AHTPDB), and their antidiabetic activity was verified using the BIOPEP database. Peakview software was used to evaluate the produced mass spectra and determine the peptide sequences.

### 2.11. Fourier Transform Infrared Spectroscopy (FTIR)

At room temperature, the absorption spectra were generated using an FTIR (Bruker, Invenio-R, Billerica, MA, USA) instrument with a resolution of 4 cm-1 and a wavelength range of 400 to 2200 cm^−1^. A loading pressure was used to bring samples towards a diamond crystal. The spectrum of an empty cell was used to generate the background. The steps outlined by Leon-Lopez et al. [38] were adhered to in order to analyze the outcomes with OPUS software (Bruker, Invenio-R, Billerica, MA, USA).

### 2.12. Cell Culture

The National Cell Science Center in Pune provided the RAW 264.7 cells for analysis (Maharashtra, India). Phosphate-buffered saline (PBS) and Dulbecco’s modified Eagle’s medium (DMEM) were purchased from Lonza, Bioscience, Switzerland, and P/S (Gibco (Thermofisher Scientific, Waltham, MA, USA). MP Biomedicals donated fetal bovine serum (FBS). Lipopolysaccharide (LPS) was purchased (China) from Cusabio Biotech. Tumor necrosis factor-alpha (TNF-α), interleukin-6 (IL-6), amp, and the interleukin-1 beta (IL-1β) ELISA kits were bought from Elabscience (Houston, Texas, USA).

#### 2.12.1. Cell Viability

Frozen stock culture of RAW 264.7 cells were activated in DMEM with 10% fetal bovine serum and 1% penicillin/streptomycin (P/S) in a 25 cm^2^ tissue culture flask and incubated in a humidified incubator under 5% CO_2_ at 37 °C. After a confluent layer of cells was formed, the cells were collected by gentle scrapping and then centrifuged and suspended in fresh DMEM containing FBS and P/S, seeded at a density of 1 × 10 ^5^ cells in each well of a 96-well plate and incubated as mentioned above for another 24 h. The confluent cells were treated with fresh DMEM consisting of camel and buffalo milk fermented with KGL4 + WBS2A at 2, 1, 0.5, and 0.25 mg/mL for 24 h. An MTT (03-(4,5-dimethylthiazol-2-yl)-2,5-diphenyltetrazolium bromide) assay was carried out using the protocol provided by Khare et al. [39]. The MTT was added to a final concentration of 0.5 mg/L and incubated at 37 °C under 5% CO_2_ in the dark for 4 h. The formazan crystals that formed were dissolved in 100 µL of DMSO, and the optical density was determined using a microplate reader at 570 nm (M200 PRO, Tecan Life Science, Seestrasse, Männedorf, Switzerland).

#### 2.12.2. Anti-Inflammatory Activity on LPS-Activated Macrophages

A 48-well plate containing (2 × 105 cells) RAW 264.7 cells was seeded before a 24 h incubation period. Additionally, 1 μg/mL of LPS and KGL4+WBS2A that had been fermented with camel and buffalo milk was administered to the confluent macrophages. Additionally, the cells were cultured for 16 h in a humidified CO2 incubator before the nitrite concentration was calculated using the Griess reagent method [39].

#### 2.12.3. Measurement of TNF-α, IL-6, and IL-1β Cytokines

Using commercially available ELISA kits and following the manufacturer’s instructions, the concentrations of TNF-α, IL-6, and IL-1β were found in the cell supernatants collected after interventions (Elabscience, Houston, Texas, USA).

### 2.13. Molecular Docking

The peptide sequences in our study’s analysis had their 3D structures predicted using PEPstrMOD [40]. Using the PDB IDs 3BAI, 3CTT, 1F6W, and 1O8A, respectively, crystallographic 3D structures for the human bile salt-activated lipase (hBAL), human maltase-glucoamylase (hMGA), human pancreatic alpha-amylase (hPAM), and human angiotensin-converting enzyme (hACE) were retrieved. The structures were chosen based on the PDB’s highest resolution. Protein macromolecules were enhanced by utilizing AutoDock 4.2.6 (La Jolla, California 92037 USA) to remove water molecules and heteroatoms and to substitute them with polar hydrogens and Kollman charges. The ligand binding sites in the protein structures were predicted using the PrankWeb web server [41]. Molecular docking was conducted using the top binding cavity and the HADDOCK 2.4 online tool [42]. The docking complexes for each peptide ligand (EAIR) were assessed using the HADDOCK score, which is calculated as the product of the following energies: 1.0 van der Waals energy (Evdw) + 0.2 electrostatic energy (Eelec) + 1.0 desolvation energy (Edesol) + 0.1 restraint violation energy. Better binding affinity prediction is indicated by a higher negative HADDOCK score. The docked complexes (European Bioinformatics Institute) were visualized using LigPlot+ and Discovery Studio Visualizer 2020 (Biovia, San Diego, USA).

### 2.14. Statistical Analysis

The data that were obtained were examined using the methods described by Steel and Torrie [43]. A 5.0% level of significance was used to examine the significance of each parameter’s impact on a particular attribute. Five percent was determined as the threshold for statistical significance. A one-way ANOVA was used to compare several groups, followed by Tukey’s post hoc analysis. GraphPad Prism 8.0 Software Inc. (La Jolla, CA, USA) was used to analyze the data. *p* ≤ 0.05 was chosen as the cutoff point for statistical significance. Three replications were performed for all the activities.

## 3. Results and Discussions

### 3.1. ACE Inhibitory and Anti-Diabetic Activities of Fermented Camel and Buffalo Milk

**Figure 1:**
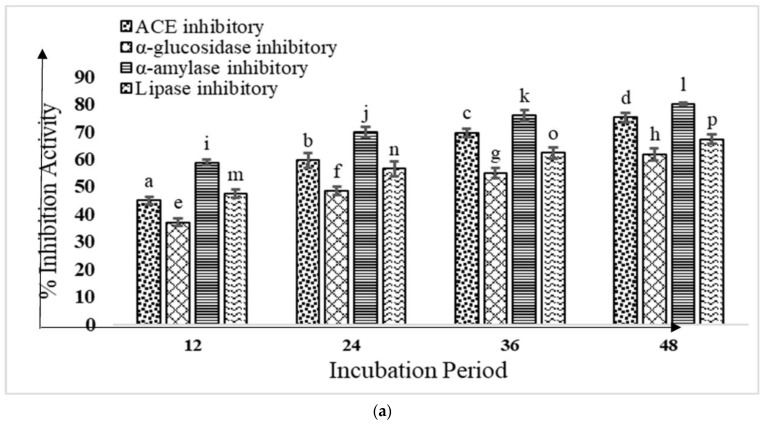

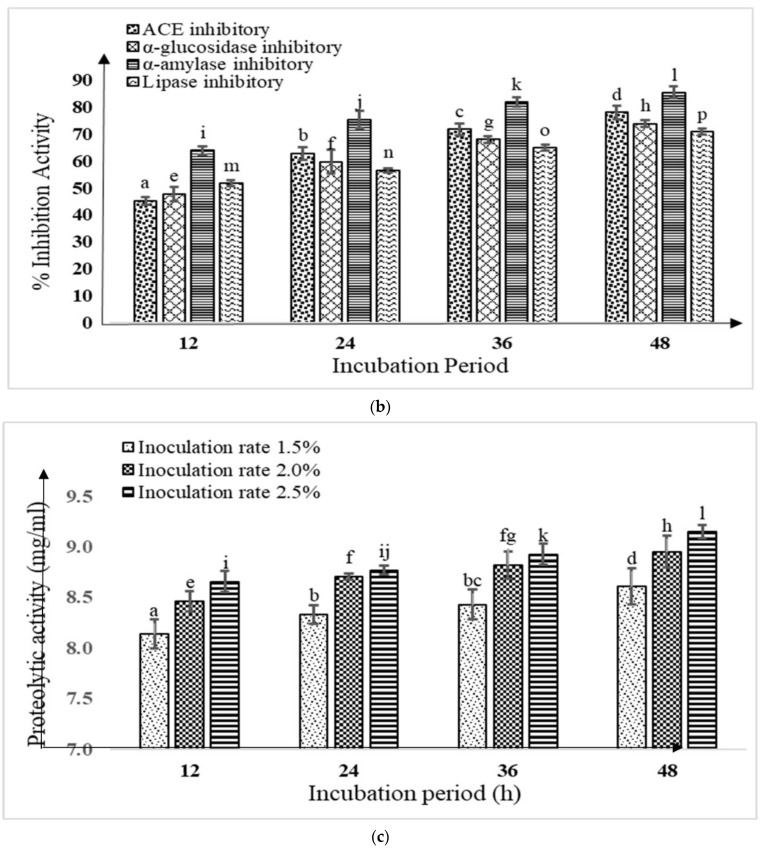

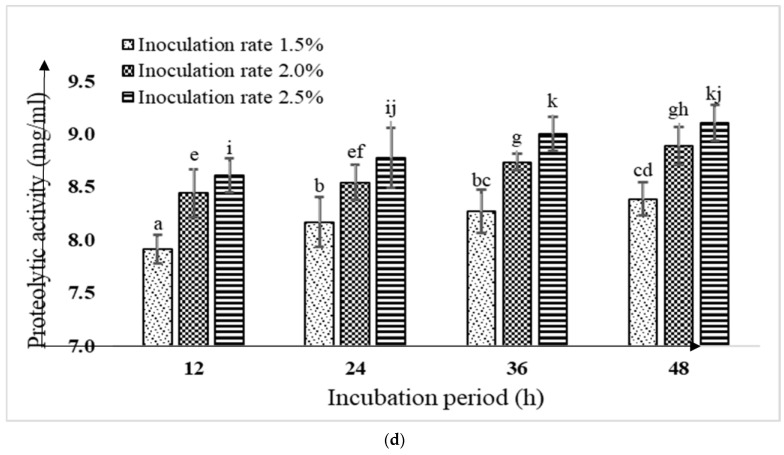
(**a**) Fermented buffalo milk’s ACE inhibitory and anti-diabetic activity (%); mean ± SD of three replicate studies (n = 3); values with different superscripts differ significantly (*p* < 0.05). (**b**) Fermented camel milk’s ACE inhibitory and anti-diabetic activity (%); mean ± SD of three replicate studies (n = 3); values with different superscripts differ significantly (*p* < 0.05). (**c**) Proteolytic activity (mg/mL) of buffalo milk fermented using KGL4 + WBS2A; mean ± SD of 3 replicates; each sample was subjected to a total of 3 replicates; values with different superscripts differ significantly (*p* < 0.05). (**d**) Proteolytic activity (mg/mL) of camel milk fermented using KGL4 + WBS2A; mean ± SD of 3 replicates; each sample was subjected to a total of 3 replicates; values with different superscripts differ significantly (*p* < 0.05).

To estimate the anti-diabetic effects and ACE inhibitory properties of fermented buffalo and camel milk, the KGL4 strain’s potential in conjunction with yeast WBS2A was assessed (Figure 1a,b). The milk and incubation times varied significantly (*p* < 0.05), and as the incubation times increased, the inhibitory and anti-diabetic effects of ACE were subsequently also elevated. The maximum ACE inhibitory, lipase inhibitory, alpha-glucosidase inhibitory, and alpha-amylase inhibitory activities of the fermented buffalo milk (FBM) (75.25 ± 1.72, 61.79 ± 2.14, 80.09 ± 0.51, and 67.29 ± 1.75) and fermented camel milk (FCM) (77.96 ± 2.61, 73.85 ± 1.19, 85.37 ± 2.15, and 70.86 ± 1.02), compared to the other incubation times at 37 °C, were seen at 48 h.

Both the camel and the buffalo milk exhibited a substantial increase (*p* < 0.05) in ACE enzyme inhibition with increasing incubation times, which Shukla [44] had previously noticed. According to Shukla [44], camel milk fermented with *L. fermentum* (M4), *L. paracasei* (M11), *L. rhamnosus* (M9), *L. fermentum* (KGL4), and *L. plantarum* (KGL3A) showed ACE inhibitory activity in the range of 43.29–78.33% from 0 to 48 h of incubation. M11 had the highest ACE inhibitory activity at 48 h (76.33%), followed by KGL3A (77.53%), KGL4 (76.08%), and M4 (70.87%), while M9 demonstrated the lowest ACE inhibitory activity (70.56%) at 37 °C. The efficacy of fermented bovine and camel milk in inhibiting α-amylase was evaluated by Ayyash et al. [45]. Both camel and bovine milk were fermented separately using three probiotic strains from camel milk: *Lb. plantarum*-KX881772 (Lp.K772), *Lb. reuteri*-KX881777 (Lr. K777), *Lb. plantarum*-KX881779 (Lp.K779), and a control strain, *Lb. plantarum* DSM2468. The fermentation of both the bovine and the camel milk was performed using LAB cultures for 24 h at 37 °C, and the cultures were then stored for 21 days at 4 °C to analyze their ability to block alpha-amylase. With the exception of camel milk, which Lp.K772 fermented, they reported > 34% amylase inhibition in both milk types throughout storage. Camel milk fermented with Lr.K777 showed the highest inhibition > 34% (51%) among all the cultures, followed by Lp.K779 (46%). On the 28th day of storage, Lp.K772 showed the lowest alpha-amylase inhibition (34%), while Lp.DSM showed the highest (38%). Lr.K777 culture also showed the maximum inhibition in fermented bovine milk, followed by Lp.K779, Lp.DSM, and Lp.K772 with the lowest inhibition.

Jafar et al. [46] examined the degree to which camel whey protein hydrolysates inhibited pancreatic lipase activity (CWPHs). Camel whey proteins that had undergone hydrolysis by gastric and pancreatic enzymes displayed a noticeably greater lipase inhibitory effect (*p* < 0.05) than the camel whey proteins that had not. After 3 and 6 h of hydrolysis time, the most potent pancreatic lipase inhibitory effect was seen in pepsin-produced hydrolysates (*p* < 0.05). After 3 and 6 h of hydrolysis, trypsin-generated CWPHs showed the lowest levels of inhibition (34.89 ± 1.08% and 25.97 ± 2.72%). Ayyash et al. [47] made low-fat Akawi cheese by mixing camel and bovine milk at the ratios of 100:0, 85:15, or 70:30 and allowing it to ripen for 28 days. Then, at day 0 and after 28 days, they determined the alpha-amylase inhibition activity in the WSE, excrement, and bio-accessible fractions from various cheeses. After 28 days of being maintained at 4 °C, the camel and cow milk combinations were used to create cheese. On day 28, compared to the cheese produced only with bovine milk, the WSE from cheeses made with 30% camel milk and 15% camel milk showed greater (*p* < 0.05) alpha-amylase inhibitory rates (>50%). This study confirmed our findings regarding the growing alpha-amylase inhibition activity after fermentation and the increasing alpha-amylase inhibition with the incubation durations.

### 3.2. Optimization for the Development of Peptides in Fermented Camel and Buffalo Milk

With increasing inoculation rates (1.5%, 2.0%, and 2.5%) and incubation times (12, 24, 36, and 48 h), KGL4 + WBS2A fermented buffalo and camel milk considerably (*p* < 0.05) increased the proteolytic activity, despite the fact that there were no statistically significant connections found between the inoculation rate and incubation period (Figure 1c,d). When milk was inoculated at a rate of 2.5%, the highest level of proteolysis activity was seen after 48 h.

Our findings show that as the incubation periods and inoculation rates increased in both milks, proteolytic activity increased as well. Dharmisthaben et al. [48] studied the proteolytic activity of camel milk fermented with *Lactobacillus plantarum* KGL3A at various incubation periods (0, 12, 24, and 48 h) and at varied inoculum rates (1.5%, 2.0%, and 2.5%). The proteolytic action of KGL3A considerably increased during the incubation durations of 0, 12, 24, and 48 h (*p* < 0.05), despite the fact that there were no significant differences in the interactions between the incubation length and the inoculum rate or inoculation rates. At a 2.5% inoculation rate, the maximum proteolysis (7.84 mg/mL) was observed after 48 h. This study confirms our findings that proteolytic activity peaked at 48 h and at the 2.5% inoculation rates, increasing with the incubation lengths and inoculation rates.

In heat-treated camel milk fermented with Lb. rhamnosus MTCC 5945, the growth conditions required for the peptide production were discovered by Solanki and Hati [49]. (NS4). The proteolytic activity of camel milk that had undergone fermentation was calculated for a variety of incubation times (0, 3, 6, 9, and 12 h) and inoculation rates (1%, 1.5%, and 2.0%). The range of their measurement for OPA absorbance was 0.526 to 1.024 (OD at 410 nm). Additionally, they observed that the proteolytic activity was boosted by the incubation durations and that this activity varied greatly (*p* < 0.05), which is consistent with the findings of Solanki et al. [31]. For all the inoculum rates (1%, 1.5%, and 2%), the lactic culture displayed noticeably higher levels of proteolytic activity after 12 h compared to the other incubation times. It was also shown that proteolytic activity peaked after 12 h and at a 2% inoculum rate when compared to different ratios of inoculation rates and incubation times. This investigation supports our results showing that incubation time and inoculation rate enhanced proteolytic activity. Therefore, the highest proteolytic activity of our experiment was identified at a 2.5% inoculation rate after 48 h.

### 3.3. SDS-PAGE Analysis of WSE from Fermented Camel and Buffalo Milk

**Figure 2:**
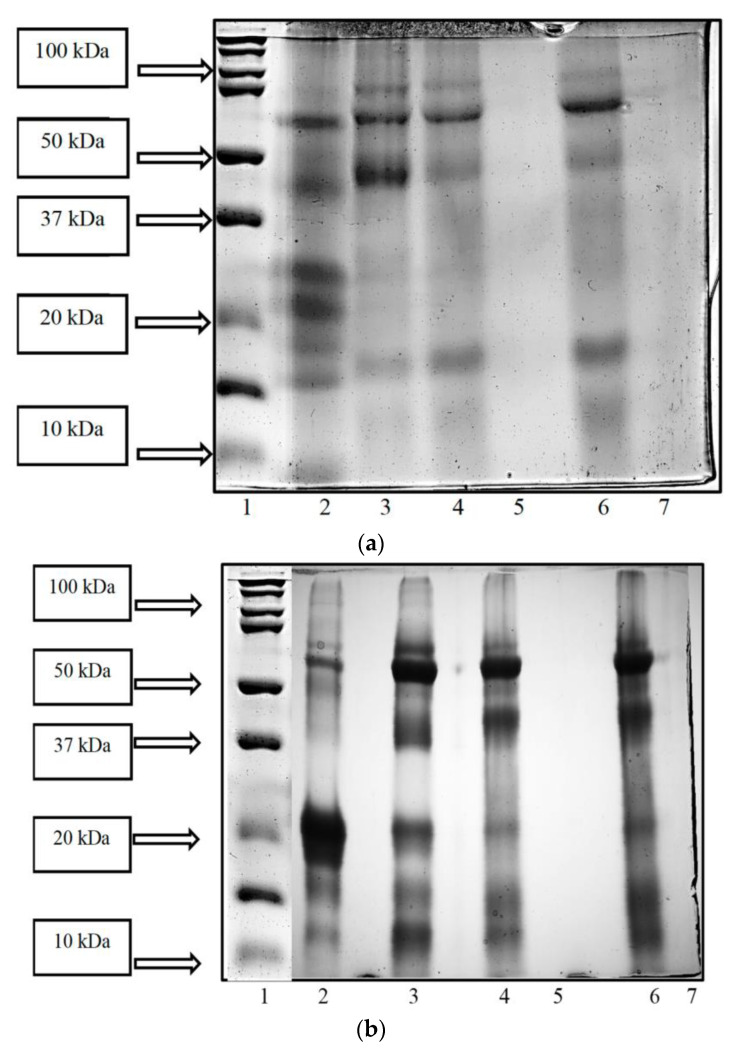
(**a**) SDS-PAGE-revealed protein and peptide profile of buffalo milk fermented with KGL4 + WBS2A (1: protein ladder; 2: buffalo milk sample (unfermented); 3: buffalo milk sample (fermented); 4: >3 kDa sample; 5: <3 kDa sample; 6: <10 kDa sample; 7: >10 kDa sample). (**b**) SDS-PAGE revealed protein and peptide profile of camel milk fermented with KGL4 + WBS2A (1: protein ladder; 2: camel milk sample (unfermented); 3: camel milk sample (fermented); 4: >3 kDa sample; 5: <3 kDa sample; 6: <10 kDa sample; 7: >10 kDa sample).

Using a low molecular protein ladder, SDS-PAGE analysis of WSEs made from fermented buffalo and camel milk was conducted (M W. 10–250 kDa). When compared to the milk samples that had undergone fermentation, unfermented camel and buffalo milk provided the majority of natural protein bands on SDS-PAGE. Protein bands with sizes between 10 and 100 kDa and 10 and 75 kDa were present in the samples of camel and buffalo milk that had not undergone fermentation, However, all of the fermented samples contained bands that were found in sizes ranging from 10 to 75 kDa. However, in the permeate samples, no protein bands were present (Figure 2a,b).

Shukla [44] investigated the WSEs produced by *L. plantarum* (KGL3A), *L. paracasei* (M11), and *L. fermentum* (KGL4) fermented camel milk. The camel milk that was not fermented had the largest protein bands on SDS-PAGE compared to the fermented camel milk. This illustrated the powerful proteolytic activity of the LAB in camel milk. Protein bands between 10 and 130 kDa were identified in camel milk that was not fermented, whereas bands between 10 and 100 kDa were identified in all the samples of fermented camel milk. However, no bands were found in the permeate of KGL3A or M11, but they were found in the case of the KGL4 permeate. SDS-PAGE was used by Gonçalves et al. [50] to compare the electrophoretic profiles of proteins and peptides from mozzarella cheeses manufactured from cow, buffalo, or combinations of those milks. Cheeses branded “reference treatment buffalo” or “reference treatment cow” were made using both buffalo and cow milk (RTB and RTC, respectively). The buffalo milk was combined with 2.5%, 5.0%, 10%, 20%, 30%, 40%, or 50% cow milk to create standardized cheeses. When they compared the RTB and RTC samples, they discovered minor differences in the s-CN structure as well as differences in the -CN’s electrophoretic mobility. The structure and mobility of the αs-CN and β-CN both changed somewhat as the amount of cow milk increased. More peptides were found in the cheese after it had been refrigerated for 20 days, and the fractions αs-CN and β-CN were degraded, particularly in the areas below β-CN in the RTB sample.

### 3.4. Two-dimensional Gel Electrophoresis

In 2D gel electrophoresis, KGL4 + WBS2A-developed fermented buffalo milk protein was identified in 15 areas and fermented camel milk protein in 20 spots. The protein spots in the 2D gel electrophoresis varied from 20 to 75 kDa (Supplementary Figure S1a,b). In camel milk fermented by KGL3A, Dharmisthaben et al. [48] also carried out 2D gel electrophoresis; a total of 19 spots were found. The protein’s size fluctuated from approximately 42 kDa to 124 kDa during the 2D gel electrophoresis. After RP-LC/MS of the digested 2D spots, they discovered novel antioxidative sequences (LLILTC, AVALARPK, YPLR, LSSHPYLEQLYR, and TQDK).

Specifically focusing on k-casein fractions in order to develop a protein profile, Dettori et al. [51] used 2D electrophoresis to assess buffalo milk samples with considerably different cheese yield values. Four buffaloes were chosen from a sample of 135 animals for the proteomic studies, two of which had good cheese yields while the other two had low yields. Alpha-lactoalbumin, beta-lactoglobulin, k-casein, β-casein, αs1-casein, and αs2-casein were identified as the six primary spots in 2D gels. The four k-casein spots, which varied in the amount of phosphorylation and glycosylation, were determined to be the most obvious variances in the 2D gels when comparing buffaloes with high vs. low cheese yield.

### 3.5. Evaluation of ACE Inhibitory, Anti-Diabetic, and Proteolytic Activities of Ultra-Filtered Fractions of WSE

**Figure 3:**
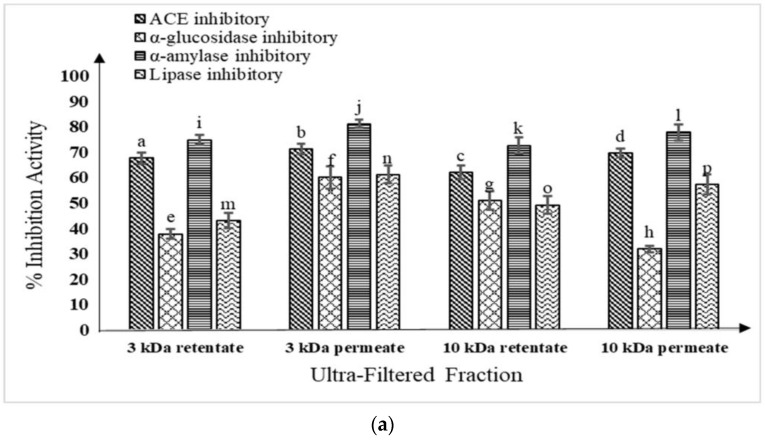

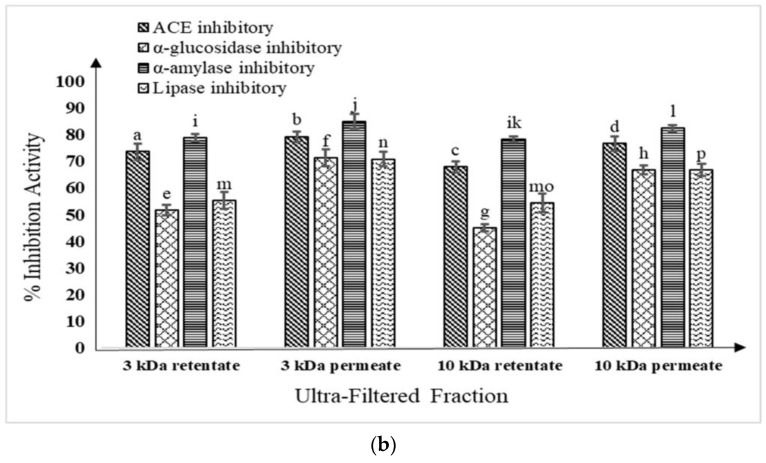
(**a**) Fermented buffalo milk (M11 + WBS2A) ultra-filtered fractions (<3 kDa, >3 kDa, <10 kDa, and >10 kDa) ACE inhibitory and anti-diabetic activities; mean ± SD of three replicate studies (n = 3); values with different superscripts differ significantly (*p* < 0.05). (**b**) Fermented camel milk (M11 + WBS2A) ultra-filtered fractions (<3 kDa, >3 kDa, <10 kDa and >10 kDa) ACE inhibitory and anti-diabetic activities; mean ± SD of three replicate studies (n = 3); values with different superscripts differ significantly (*p* < 0.05).

It was demonstrated that ultra-filtered portions of fermented buffalo and camel milk contain ACE inhibitory, anti-diabetic, and proteolytic properties, as seen in Figure 3a,b. During fermentation, the peptide fractions of buffalo (Supplementary Figure S2a–f) and camel milk (Supplementary Figure S2g–l) were visible in the RP-HPLC chromatograms of ultra-filtered fractions (3 and 10 kDa permeates and retentates).

The highest number of peaks were found in the 3 kDa permeate chromatogram of fermented buffalo milk (58) and the 3 kDa retentate fraction of fermented camel milk (54) with, correspondingly, retention times ranging from 26.17 to 54.74 and 24.79 to 54.29 (Table 1).

The ACE-I activity of isolated and digested buffalo milk casein with various digestive enzymes was assessed by Shanmugam et al. [52] (trypsin, chymotrypsin, pepsin, and their combinations). The highest level of ACE inhibition was observed in pepsin–trypsin hydrolysates (72.55 ± 2.23%/50 μg). When compared to other fractions, the ultrafiltration fraction <1 kDa fraction’s ACE-I activity was significantly different (*p* < 0.01) and had the strongest inhibition effect (83.0 ± 0.89% inhibition per 50 µg of peptides). A reduction in the hydrolysates’ molecular weight led to an increase in ACE-I activity. This investigation supports our findings, which revealed that the ACE-I activity was maximum in the <3 kDa sample, followed by the <10 kDa and the >3 kDa and was lowest in the >10 kDa sample. Lipase inhibitory peptides were isolated and purified by Kim and Lim [53] from milk that had been fermented by *L. plantarum* Q180. When the culture’s pH reached pH 4.4, *L. plantarum* 180 was added to reconstituted skim milk (10%) and then kept for incubation at 37 °C. The supernatants were then filtered through a range of molecular weight cutoff thresholds <1000 Da. After *L. plantarum* Q180 ultra-filtered fermented milk, the yield of the <1000 Da fraction and the anti-lipase activity were 46.83% and 96.10%, respectively. These findings suggest that low-molecular-weight peptides have stronger lipase-inhibiting effects.

### 3.6. RP-LC/MS Characterization and Identification of Purified Peptides

Peptide ranking values greater than 0.45 were found in nine sequences from fermented buffalo milk and five sequences from fermented camel milk. Mass spectrometry analysis was used to extract sequences from the 2D PAGE study. Different peptide sequences from fermented camel and buffalo milk were confirmed using Peakview software (Table 2). The AHTPDB (Supplementary Table S1a,b) and BIOPEP (Supplementary Table S1c,d) [6,54,55,56,57,58,59,60,61,62,63,64,65,66,67,68,69,70,71,72,73,74,75,76,77,78] databases were examined against a variety of di-, tri-, and tetrapeptide sequences to identify ACE inhibitory and anti-diabetic peptides, respectively. Supplementary Figure S3a–d, respectively, display the total ion chromatograms of a 2D spot from fermented buffalo and camel milk.

According to our research, several peptide sequences with high peptide ranking scores (>0.450) were found in the 2D PAGE of fermented buffalo and camel milk. These sequences include SCQAQPTTMTR, EMPFPK, TTMPLW, HPHPHLSFMAIPPK, FFNDKIAK, ALPMHIR, IPAVFK, LDQWLCEK, and AVPYPQR from the fermented buffalo milk and TDVMPQWW, EKTFLLYSCPHR, SSHPYLEQLY, IDSGLYLGSNYITAIR, and FDEFLSQSCAPGSDPR from the fermented camel milk.

### 3.7. FTIR (Fourier Transform Infrared Spectroscopy)

In the samples of buffalo and camel milk, several peaks between the spectral range of 400 and 2000 cm^−1^ were seen. Samples of fermented buffalo and camel milk revealed higher peaks in the range of 1800 to 400 cm^−1^. The FTIR spectra showed that the unfermented sample had distinct peaks in the range of 516.75 cm^−1^, 822.25 cm^−1^, 1036.88 cm^−1^, 1120.08 cm^−1^, and 1628.67 cm^−1^ from the fermented buffalo milk and 512.80 cm^−1^, 1234.85 cm^−1^, 1541.59 cm^−1^, and 1628.81 cm^−1^ from the fermented camel milk (Supplementary Figure S4a,b).

In order to compare distinct milk samples, Ravinder et al. [79] used microscopic examination and spectroscopy. Buffalo milk flakes’ FTIR spectra showed absorption bands at 3435, 1641, and 1384 cm^−1^. Similar absorption bands at 3450, 1640, and 1384 cm^−1^ were seen in cow milk flakes, indicating the presence of the functional groups O-H, C=C, and C-H. The existence of OH and CH as functional groups was shown by the presence of two absorption bands in goat milk flakes at 3443 and 1384 cm^−1^. However, absorption bands at 1077, 1384, 1644, and 3418 cm^−1^ were visible in human milk flakes. Donkey milk exhibited an absorption band at 1092, 1384, 1640, and 3417 cm^−1^, just like human milk does, demonstrating the presence of the functional groups OH, C=C, C-H, and C=O. By analyzing these FTIR spectra, researchers discovered that the common functional groups in five distinct milk flakes are, in order, OH, C=C, CH, and C=O.

### 3.8. KGL4 + WBS2A Did Not Alter the Cell Viability in RAW 264.7 Cells

**Figure 4:**
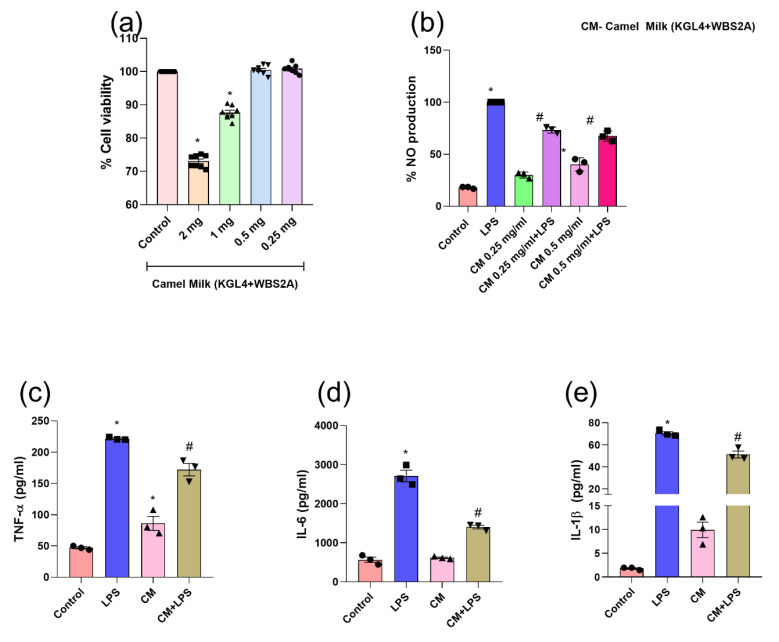
Effect of the fermented camel milk (KGL4 + WBS2A) on (**a**) cell viability (MTT test); (**b**) NO productions; (**c**) tumor necrosis factor-alpha; (**d**) interleukin-6; and (**e**) interleukin-1 beta production in the supernatants of LPS-stimulated murine macrophage (RAW 264.7) cells. Data were analyzed using one-way ANOVA and then Tukey’s post hoc test. Values were presented as mean ± SEM; (n = 3) * vs. control, # vs LPS; LPS—lipopolysaccharide; CM—fermented camel milk (KGL4 + WBS2A) (0.25 mg/mL).

**Figure 5:**
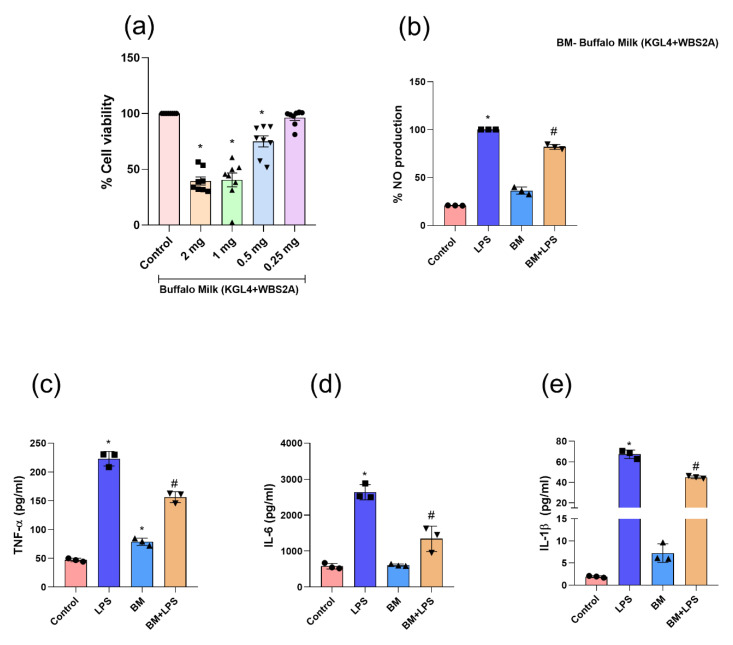
Effect of the fermented buffalo milk (KGL4 + WBS2A) on (**a**) cell viability (MTT test); (**b**) NO productions; (**c**) tumor necrosis factor-alpha; (**d**) interleukin-6; and (**e**) interleukin-1 beta measured in the supernatants of LPS-stimulated (RAW 264.7) cells. Data were analyzed using one-way ANOVA and then Tukey’s post hoc test. Values were presented as mean ± SEM; (n = 3). * vs control; # vs LPS; LPS—lipopolysaccharide; BM—fermented buffalo milk (KGL4 + WBS2A) (0.25 mg/mL).

The cytotoxic effect of fermented camel and buffalo milk on RAW 264.7 macrophages was investigated using the MTT assay at various KGL4 + WBS2A concentrations (2, 1, 0.5, and 0.25 mg/mL) (Figure 4 and Figure 5). The result demonstrates that there was no cytotoxicity at 0.25, 0.5, and 0.25 mg/mL in camel milk and buffalo milk, respectively (Figure 4a–e and Figure 5a–e). Additional camel and buffalo milk fermented with KGL4 + WBS2A at 0.25, 0.5, and 0.25 mg/mL concentrations was used for the NO and cytokine tests.

### 3.9. KGL4 + WBS2A Fermented Camel and Buffalo Milk Attenuates LPS-Induced NO Production in RAW 264.7 Cells

It was found that LPS stimulation resulted in a remarkable elevation of NO in LPS-treated macrophages relative to controls. This was prevented by camel milk doses of 0.25 and 0.5 mg/mL and buffalo milk doses of 0.25 mg/mL fermented with KGL4 + WBS2A (Figure 4b and Figure 5b). A significant anti-inflammatory effect in camel and buffalo milk was demonstrated by a significant decrease in LPS-induced NO production without inducing cytotoxicity.

#### 3.9.1. Analysis Cytokine in RAW 264.7 Cells

The figure illustrates the considerable increase in TNF-α, IL-6, and IL-1β levels after LPS-stimulated macrophages. The treatment with camel and buffalo milk fermented with KGL4 + WBS2A inhibited this rise in pro-inflammatory cytokines (Figure 4c–e and Figure 5c–e). An important role is played by nitric oxide (NO), a free-radical signaling molecule, in numerous biological processes, including inflammation. The upregulation of NO during inflammation is due to the oxidation of NO to nitrate and nitrite, which is measured by the Griess reagent. Therefore, by measuring the NO levels in RAW 264.7 macrophages, the study investigated the inhibitory effects of camel and buffalo milk fermented with (KGL4 + WBS2A) on LPS-induced NO generation. This increase in NO was due to the intervention of the cells with LPS which was found to be decreased in the camel and buffalo milk intervention groups. Pro-inflammatory cytokines, such as IL-1β, TNF-α, IL-6 are also essential cytokines that play a role in the inflammatory response and the beginning of inflammation [80]. TNF-α, IL-6, IL-1β, and NO were overproduced in this study as a result of LPS stimulation; however, buffalo and camel milk fermented with KGL4 + WBS2A dramatically reduced these levels. As to the reliability of our current figures, many earlier studies confirmed that different peptides could suppress NO production during inflammation [81,82,83]. These findings suggested that the extracts had anti-inflammatory effects by lowering NO synthesis in RAW 264.7 cells and that they might function as potent anti-inflammatory peptides when inflammation is present.

#### 3.9.2. Molecular Docking

**Figure 6:**
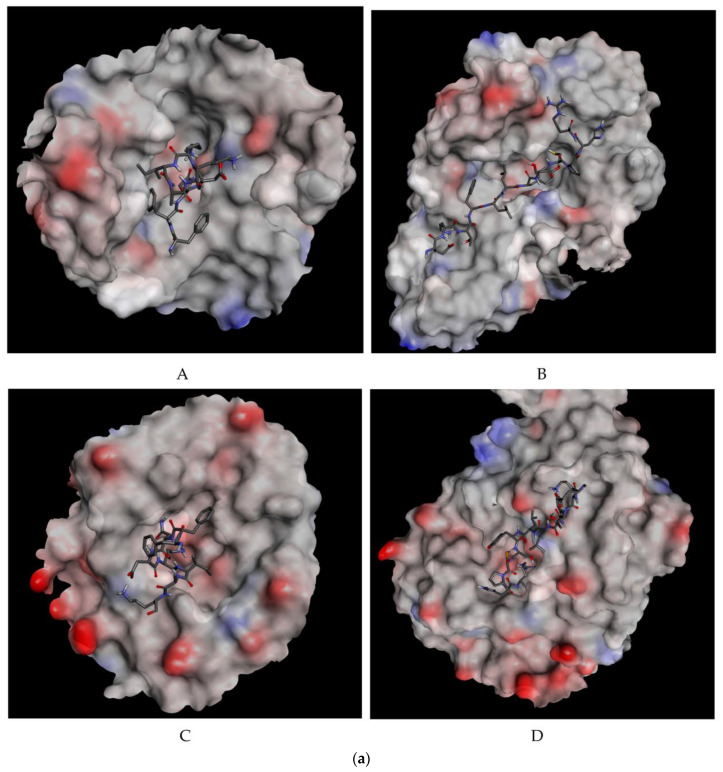

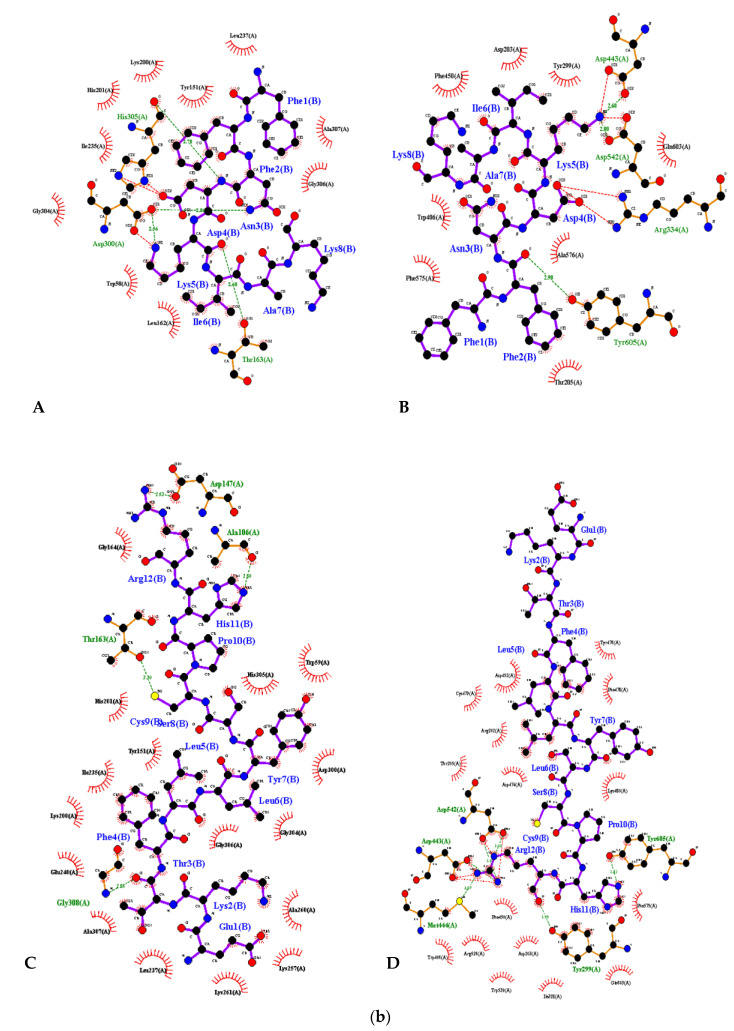
(**a**) Three-dimensional orientation of the selected peptides (represented in stick) **FFNDKIAK** and **EKTFLLYSCPHR**, respectively, in the active pockets of human pancreatic amylase (**A**,**B**) and maltase glucoamylase (**C**,**D**). (**b**) Two-dimensional representation of receptor–ligand interactions between selected peptides and receptor targets used. FFNDKIAK-3BAI (**A**); FFNDKIAK-3CTT (**B**); EKTFLLYSCPHR-3BAI (**C**); and EKTFLLYSCPHR-3CTT (**D**), 3BAI-human pancreatic alpha amylase, 3CTT-human maltase glucoamylase.

In this study, the peptides’ 3D structures were employed to analyze their binding affinities to hPAM, hMGA, and hACE binding sites. The range of the peptides’ anticipated HADDOCK scores was +4.7 to −116. The test structures had a high degree of binding affinity for the hPAM and hMGA, whereas the hACE displayed the lowest degree of binding affinity. The most significant HADDOCK scores were determined for the peptides such as FFNDKIAK (−82.7 and −81.2, respectively) and EKTFLLYSCPHR (−116.6 and −93.6, respectively). The HADDOCK values for the selected protein–peptide complexes are shown in Table 3. The intermolecular interactions supporting the docked complexes included hydrogen bonds and other hydrophobic interactions (Supplementary Table S2). The target proteins and particular peptides interacted with receptors in a visible way, as shown in Figure 6a,b. With the exception of LLNEK, which was docked against hMPO, Dharmishthaben et al. [84] found that the majority of the peptides had more torsion than the advised amount of 32. Using USFC Chimera and Autodoc Vina, different docking methods for the peptide were tested, and the model which fit the results the best had a score of roughly 7.8 kcal/mol (a measure of binding affinity). Three hydrogen bonds involving the enzyme MPO residues His261, Arg499, and Tyr500 were proposed by Chimera. The target enzyme hMPO’s active site residue for accepting protons is histidine 261, while Tyr 500 is a component of the binding sites. Additionally, the peptide formed close bonds (van der Waals overlap ≥ 0.1) with several hMPO amino acid residues, including those that are essential for catalysis, such as Gln257, Met409, and His 502.

Peptide structures were used by Ashokbhai et al. [85] to analyze their binding affinities to the HMGCS and DHFR binding sites of *E. faecalis*. The peptides had various degrees of binding affinity for both proteins, but they had a particularly high affinity for the DHFR binding site. For the peptide-Ef-HMGCS complexes, the HADDOCK scores ranged from 60.2 to 91.8, and for the peptide-Ef-DHFR complexes, they ranged from 63.1 to 93.1. They discovered that among the five peptide sequences, the complexes of the peptide FAWPQYLK-bound enzymes showed remarkable binding affinity against both the enzymes, as indicated by the HADDOCK scores. The peptide designs had a higher binding affinity for hPAM and hMGA than for hACE, according to Shukla et al. [86]. The peptides TDVMPQWW and MKFFIFTCLLAVVLAK proved to be the most effective against hPAM and hMGA based on the HADDOCK results. It was expected that the peptide EVESAEVPTENK, VALALAREK would have increased binding affinity for hACE (Figure 6b A–D).

## 4. Conclusions

The anti-diabetic and ACE inhibitory properties of camel and buffalo milk fermented by applying *Limosilactobacillus fermentum* (KGL4) and *Saccharomyces cerevisiae* (WBS2A) were significant. Compared to fermented buffalo milk (75.25%), camel milk (77.96%) had a considerably higher ACE inhibitory activity. Furthermore, fermented camel and buffalo milk showed the most anti-diabetic effects at a 2.5% inoculation rate for 48 h at 37 °C, with 61.79 and 73.85% for α-glucosidase inhibition, 80.09 and 85.37% for α-amylase inhibition, and 67.29% and 70.86% for lipase inhibition activity, respectively. From 2D gel electrophoresis, a total of nine peptide spots from fermented buffalo milk and five peptide spots from fermented camel milk were found. Using the AHTPBD and BIOPEP databases, different ACE inhibitory and anti-diabetic peptide sequences were compared to 2D PAGE-derived peptide sequences. The camel and buffalo milk showed favorable anti-inflammatory effects, as shown by a significant reduction in LPS-induced NO generation, without causing cytotoxicity. The extracts demonstrated anti-inflammatory properties by reducing NO synthesis in RAW 264.7 cells, and they may function as a potent anti-inflammatory peptide when inflammation is present.

## Figures and Tables

**Table 1:** Characterization of ultra-filtered fractions (3 and 10 kDa permeates and retentates) produced from fermented camel and buffalo milk by RP-HPLC analysis.

Sample	UF Fractions	No. of Peaks	Retention Time Range (min.)
Buffalo milk (KGL4 + WBS2A)	<3 kDa	58	26.17–54.74
	>3 kDa	52	26.58–54.77
	<10 kDa	56	22.59–53.69
	>10 kDa	54	25.37–54.74
Camel milk (KGL4 + WBS2A)	<3 kDa	34	27.19–51.27
	>3 kDa	54	24.79–54.29
	<10 kDa	25	38.27–52.52
	>10 kDa	44	25.41–54.45

**Table 2:** Amino acid sequence obtained from 2D PAGE of fermented buffalo and camel milk with peptide ranking.

Milk	Sequence	Peptide Ranking Score	Mol. wt.	Prediction	Iso-Electric Point	Net Charge at pH 7	Hydro-Phobicity	Hydro-Pathicity	Hydro-Philicity
Buffalo milk (KGL4 + WBS2A)	ALPMHIR	0.62	837.15	Non-Toxin	10.91	1.1	−0.07	0.39	−0.41
	EMPFPK	0.76	747.98	Non-Toxin	8.85	0	−0.17	−0.98	0.37
	TTMPLW	0.73	747.99	Non-Toxin	3.29	0	0.12	0.30	−1.22
	SCQAQPTTMTR	0.50	1223.53	Non-Toxin	8.6	0.9	−0.31	−0.89	−0.03
	FFNDKIAK	0.48	982.25	Non-Toxin	9.7	1	−0.17	−0.36	0.24
	HPHPHLSFMAIPPK	0.80	1609.13	Non-Toxin	9.88	1.3	−0.03	−0.42	−0.44
	IPAVFK	0.49	673.93	Non-Toxin	10.14	1	0.16	1.30	−0.55
	LDQWLCEK	0.52	1034.31	Non-Toxin	3.93	−1.1	−0.21	−0.65	0.15
	AVPYPQR	0.56	830.04	Non-Toxin	9.9	1	−0.25	−0.93	−0.16
Camel milk (KGL4 + WBS2A)	EKTFLLYSCPHR	0.49	1493.91	Non-Toxin	8.77	1	−0.22	−0.55	−0.08
	TDVMPQWW	0.74	1062.32	Non-Toxin	0.67	−1	−0.01	−0.63	−0.85
	SSHPYLEQLY	0.45	1236.49	Non-Toxin	5.1	−0.9	−0.12	−0.84	−0.49
	IDSGLYLGSNYITAIR	0.49	1756.23	Non-Toxin	6.55	0	0.00	0.36	−0.48
	FDEFLSQSCAPGSDPR	0.58	1756.08	Non-Toxin	3.54	−2.1	−0.20	−0.68	0.30

**Table 3:** HADDOCK scores for the protein–peptide complexes predicted using HADDOCK web server.

Peptide Sequences	HADDOCK Scores		
	3BAI *	3CTT *	1O8A *
Buffalo milk peptides			
**FFNDKIAK**	**−82.7+/−2.1**	**−81.2+/−2.9**	4.7 +/−8.2
LDQWLCEK	−76.0+/−0.5	−68.0+/−8.8	−16.6+/−6.4
AVPYPQR	−67.7+/−3.6	−69.9+/−1.7	17.9+/−7.4
Camel milk peptides			
**EKTFLLYSCPHR**	**−111.6+/−2.4**	**−93.6+/−2.7**	−30.1+/−10.0
SSHPYLEQLY	−83.2+/−6.0	−98.6+/−3.0	−13.4+/−1.6
IDSGLYLGSNYITAIR	−89.2+/−2.7	−73.5+/−3.5	19.1+/−10.7
FDEFLSQSCAPGSDPR	−113.0+/−2.7	−84.0+/−1.2	7.8+/−12.0

* 3BAI—Alpha amylase; 3CTT—Maltase glucoamylase; 1O8A—Angiotensin converting enzyme.

## Data Availability

Data are contained within the article.

## Supplementary Materials

The following supporting information can be downloaded at: https://www.mdpi.com/article/10.3390/foods12102006/s1, Figure S1a: 2D Gel Electrophoresis of buffalo milk fermented with KGL4 + WBS2A; Figure S1b: 2D Gel Electrophoresis of camel milk fermented with KGL4 + WBS2A; Figure S2a: RP-HPLC chromatogram of unfermented buffalo milk; Figure S2 b: RP-HPLC chro-matogram of fermented buffalo milk with KGL4 + WBS2A; Figure S2c: RP-HPLC chromatogram of 3 kDa permeate from buffalo milk with KGL4 + WBS2A; Figure S2d: RP-HPLC chromatogram of 3 kDa retentate from buffalo milk with KGL4 + WBS2A; Figure S2e: RP-HPLC chromatogram of 10 kDa permeate from buffalo milk with KGL4 + WBS2A; Figure S2f: RP-HPLC chromatogram of 10 kDa retentate from buffalo milk with KGL4 + WBS2A; Figure S2h: RP-HPLC chromatogram of fermented camel milk with KGL4 + WBS2A; Figure S2i: RP-HPLC chromatogram of 3 kDa permeate from camel milk with KGL4 + WBS2A; Figure S2j: RP-HPLC chromatogram of 3 kDa retentate from camel milk with KGL4 + WBS2A; Figure S2k: RP-HPLC chromatogram of 10 kDa permeate from camel milk with KGL4 + WBS2A; Figure S2l: RP-HPLC chromatogram of 10 kDa retentate from camel milk with KGL4 + WBS2A; Figure S3a: The total ion chromatogram of 2D spot obtained from fermented buffalo milk with KGL4 + WBS2A culture; Figure S3b: The total ion chromatogram of 2D spot obtained from fermented buffalo milk with KGL4 + WBS2A culture; Figure S3c: The total ion chromatogram of 2D spot obtained from fermented camel milk with KGL4 + WBS2A culture; Figure S3d: The total ion chromatogram of 2D spot obtained from fermented camel milk with KGL4 + WBS2A culture; Figure S4a: Fourier Transform-Infrared Spectroscopy (FTIR) of buffalo milk fermented with KGL4 + WBS2A; Figure S4b: Fourier Transform-Infrared Spectroscopy (FTIR) of camel milk fermented with KGL4 + WBS2A; Table S1a: Amino acid sequence obtained from 2D-PAGE of fermented buffalo milk (KGL4 + WBS2A) searched on AHTPBD database; Table S1b: Amino acid sequence obtained from 2D-PAGE of fermented camel milk (KGL4 + WBS2A) searched on AHTPBD database; Table S1c: Amino acid sequence obtained from 2D-PAGE of fermented buffalo milk (KGL4 + WBS2A) searched on BIOPEP database; Table S1d: Amino acid sequence ob-tained from 2D-PAGE of fermented camel milk (KGL4 + WBS2A) searched on BIOPEP database. Table S2. Details of intermolecular forces arose as a result of interaction between selected peptides and target proteins used in this study.
