# Supplementary material for: Production and Characterization of ACE Inhibitory and Anti-Diabetic Peptides from Buffalo and Camel Milk Fermented with Lactobacillus and Yeast: A Comparative Analysis with In Vitro, In Silico, and Molecular Interaction Study
Source: Foods. 2023 May 15;12(10):2006. doi: 10.3390/foods12102006 (PMC10216992; doi:10.3390/foods12102006)
Supplement: Supplementary file 1 [file foods-12-02006-s001.zip › foods-2263631-supplementary.pdf]

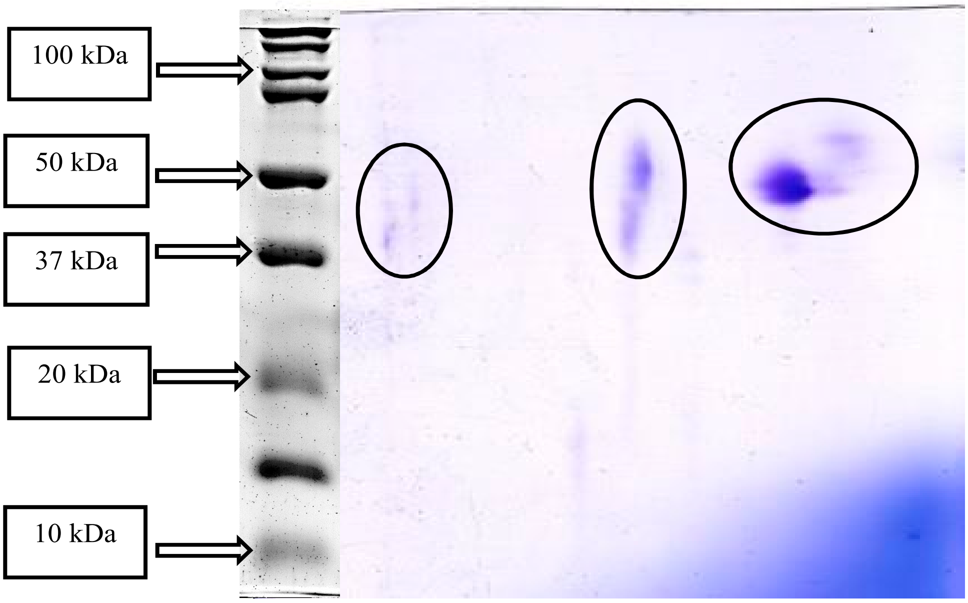

Figure S1a 2D Gel Electrophoresis of buffalo milk fermented with KGL4+WBS2A

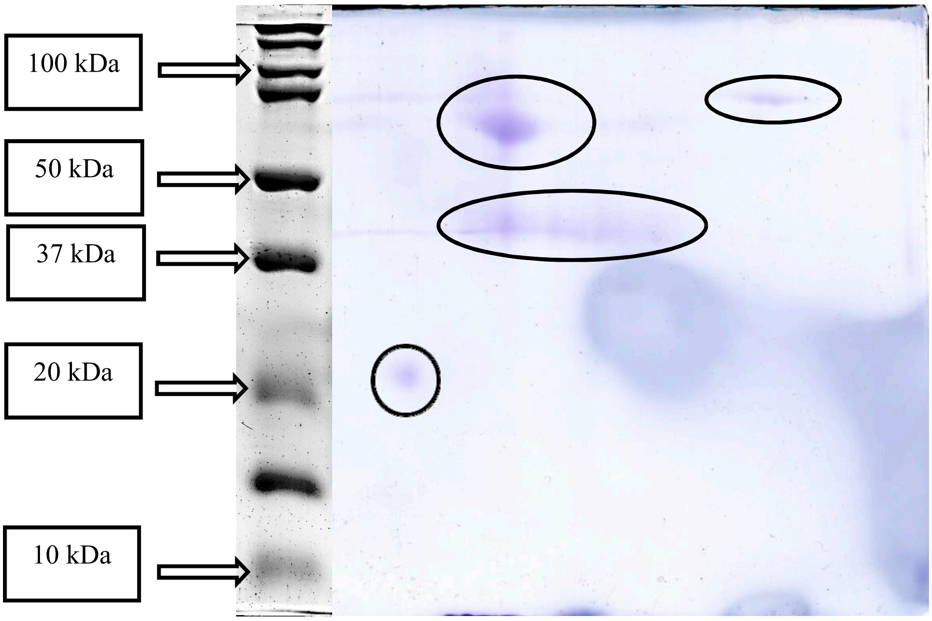

Figure S1b 2D Gel Electrophoresis of camel milk fermented with KGL4+WBS2A

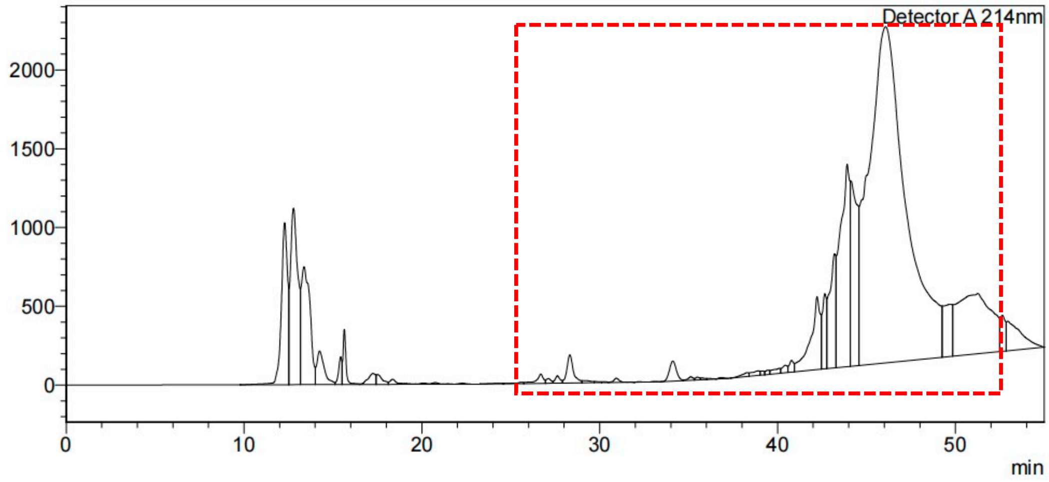

Figure S2a: RP-HPLC chromatogram of unfermented buffalo milk

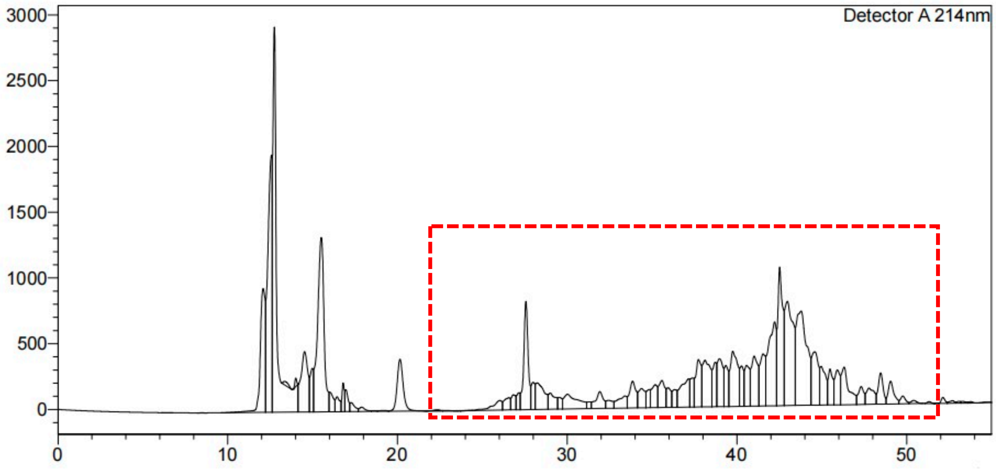

Figure S2b: RP-HPLC chromatogram of fermented buffalo milk with KGL4+WBS2A

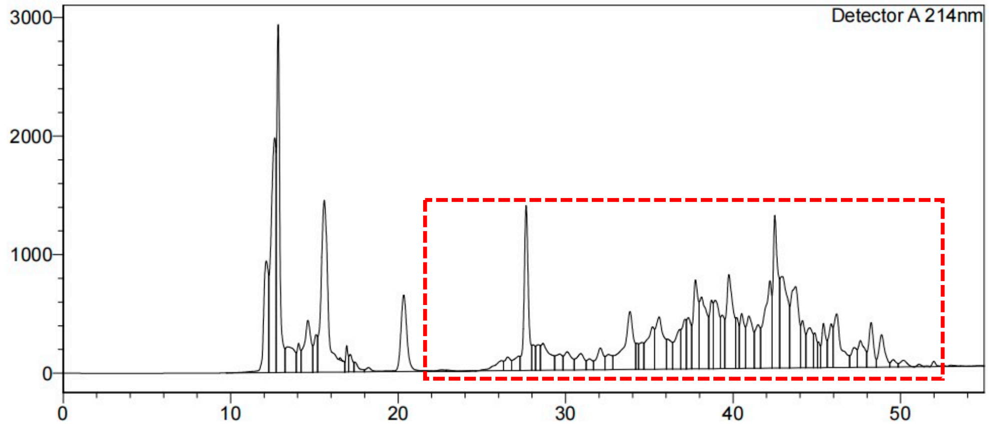

Figure S2c: RP-HPLC chromatogram of 3 kDa permeate from buffalo milk with KGL4+WBS2A

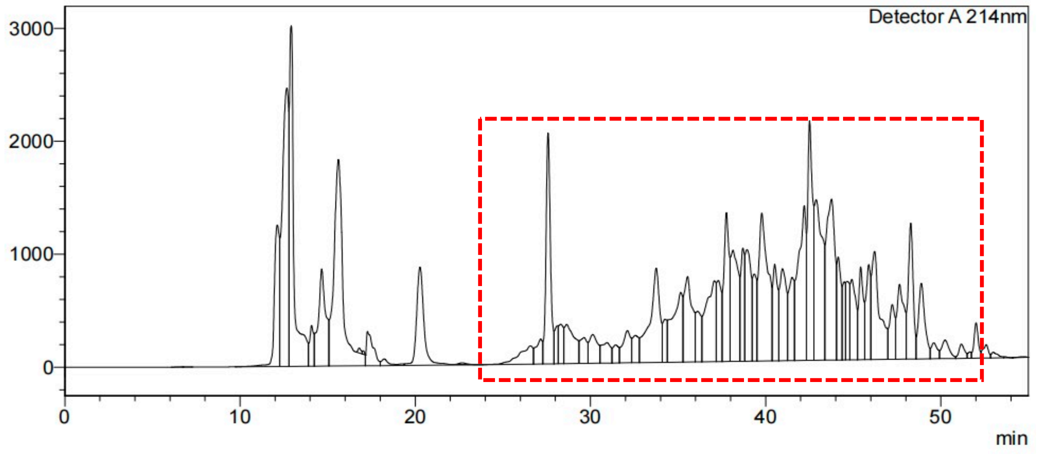

Figure S2d: RP-HPLC chromatogram of 3 kDa retentate from buffalo milk with KGL4+WBS2A

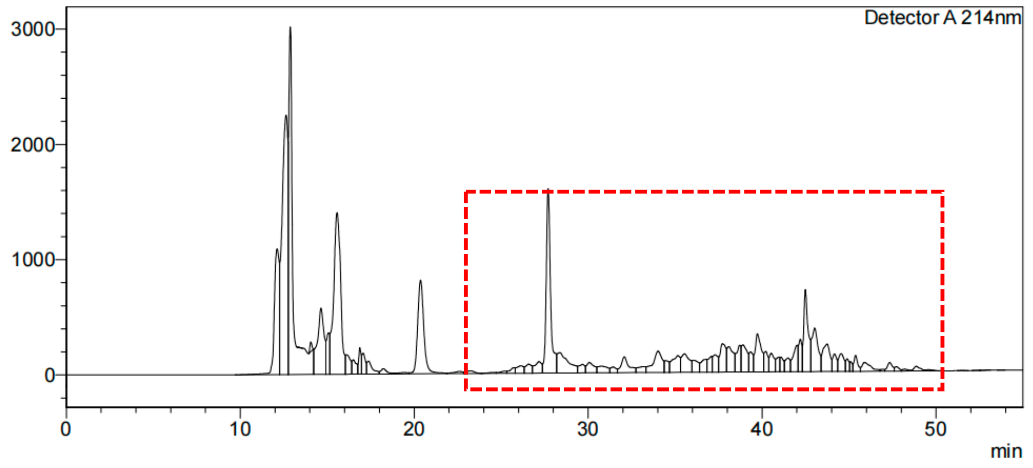

Figure S2e: RP-HPLC chromatogram of 10 kDa permeate from buffalo milk with KGL4+WBS2A

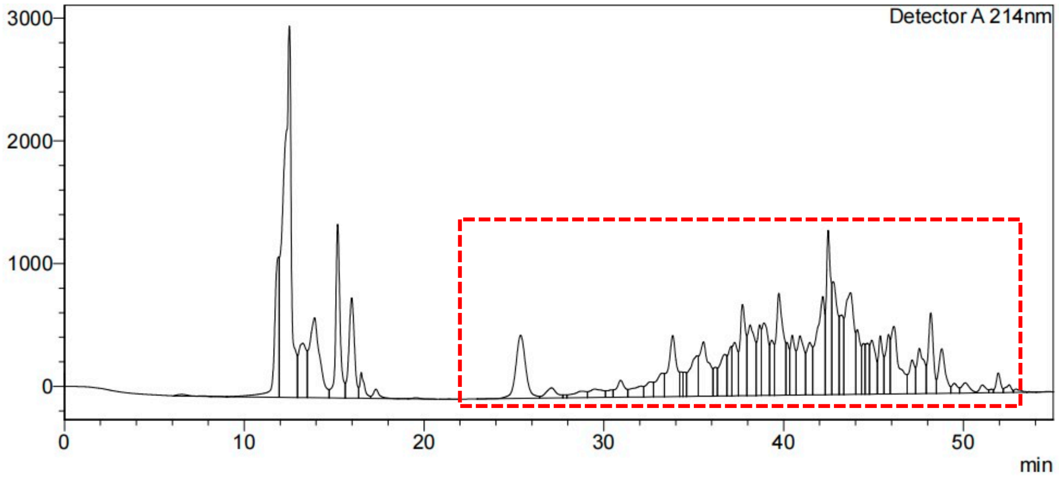

Figure S2f: RP-HPLC chromatogram of 10 kDa retentate from buffalo milk with KGL4+WBS2A

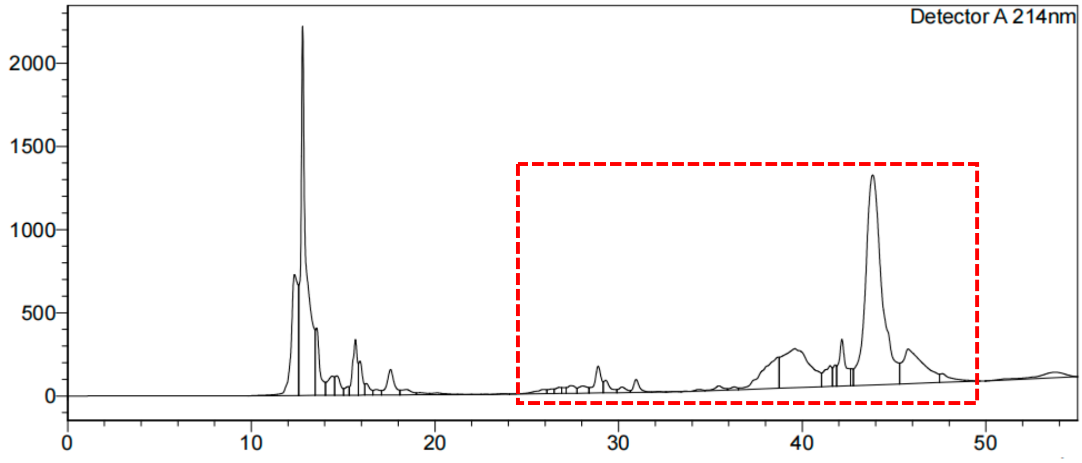

Figure S2g: RP-HPLC chromatogram of unfermented camel milk

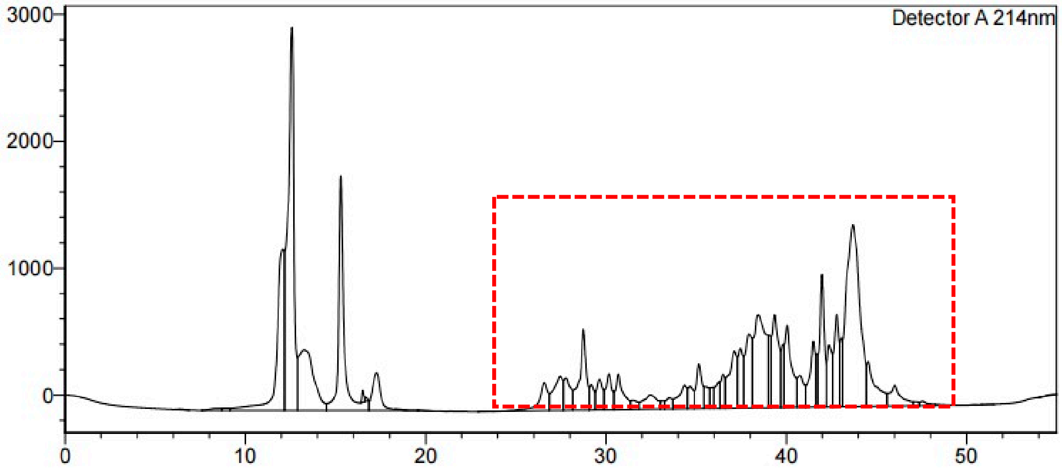

Figure S2h: RP-HPLC chromatogram of fermented camel milk with KGL4+WBS2A

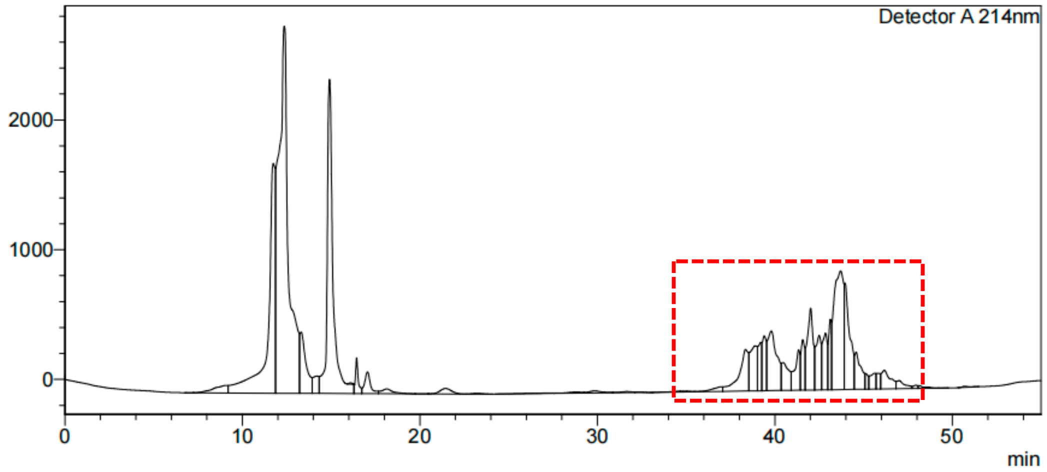

Figure S2i: RP-HPLC chromatogram of 3 kDa permeate from camel milk with KGL4+WBS2A

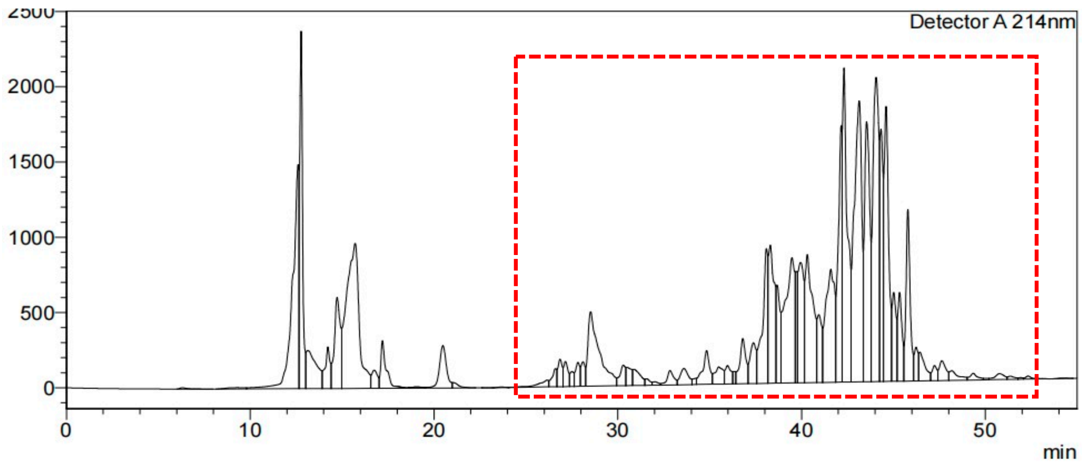

Figure S2j: RP-HPLC chromatogram of 3 kDa retentate from camel milk with KGL4+WBS2A

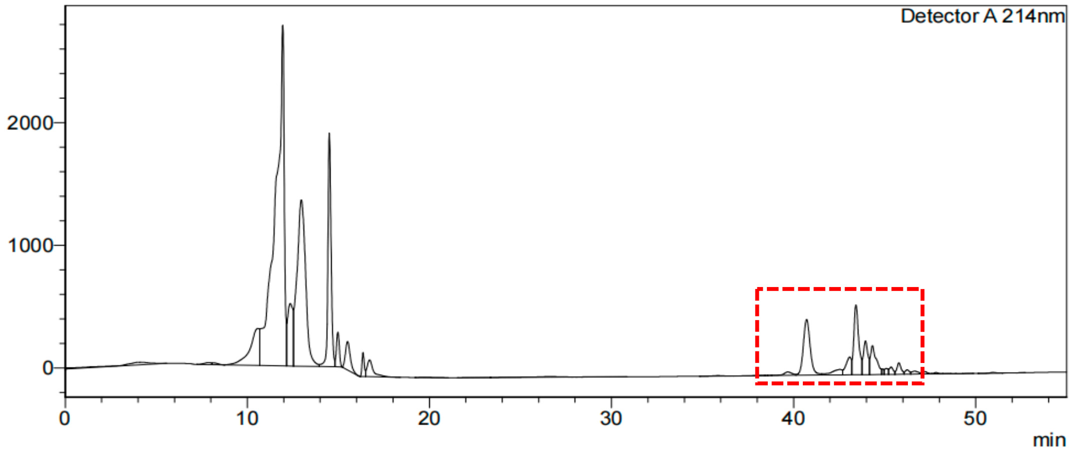

Figure S2k: RP-HPLC chromatogram of 10 kDa permeate from camel milk with KGL4+WBS2A

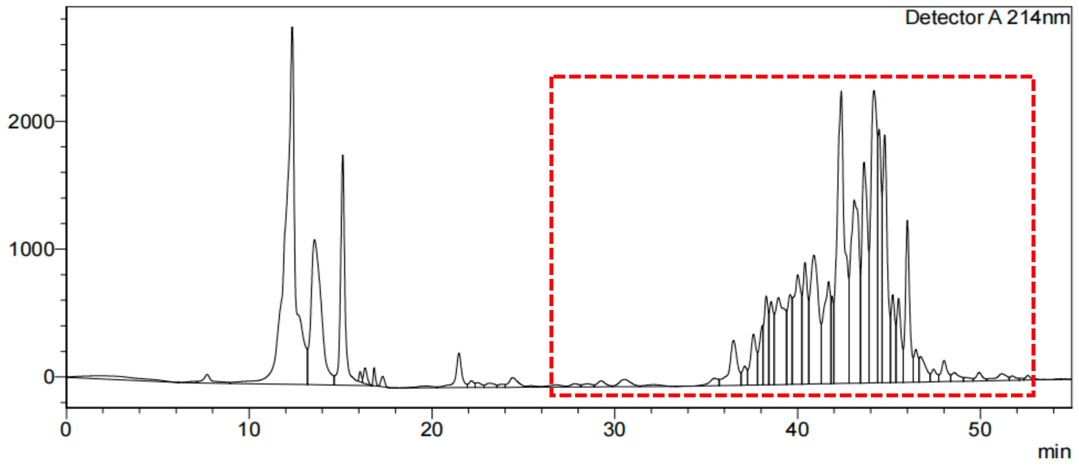

Figure S2l: RP-HPLC chromatogram of 10 kDa retentate from camel milk with KGL4+WBS2A

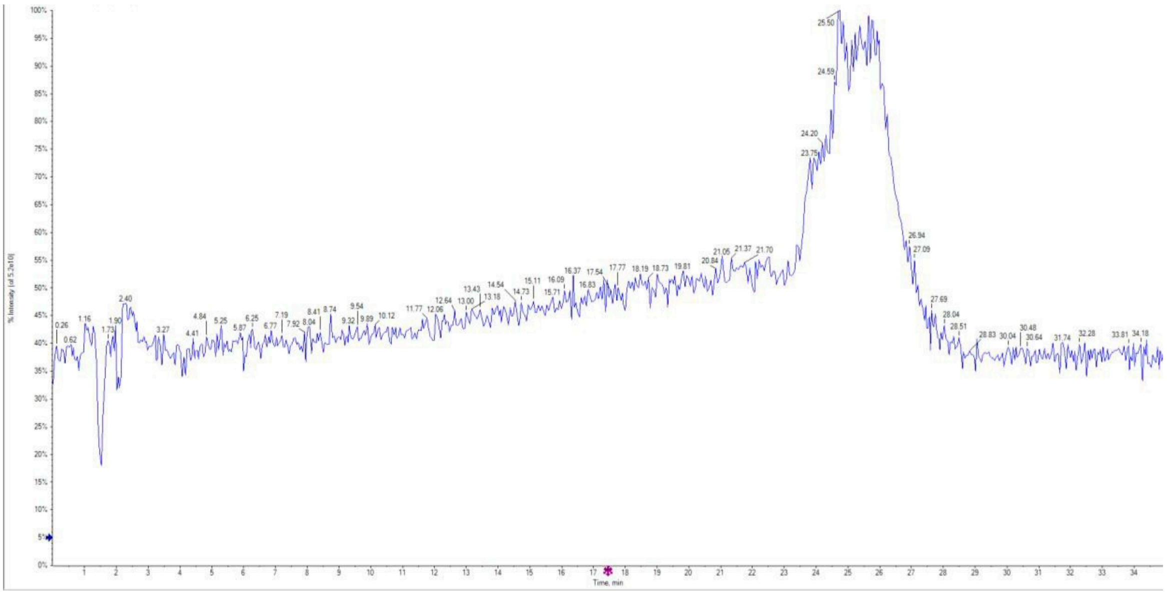

Figure S3a: The total ion chromatogram of 2D spot obtained from fermented buffalo milk with KGL4+WBS2A culture

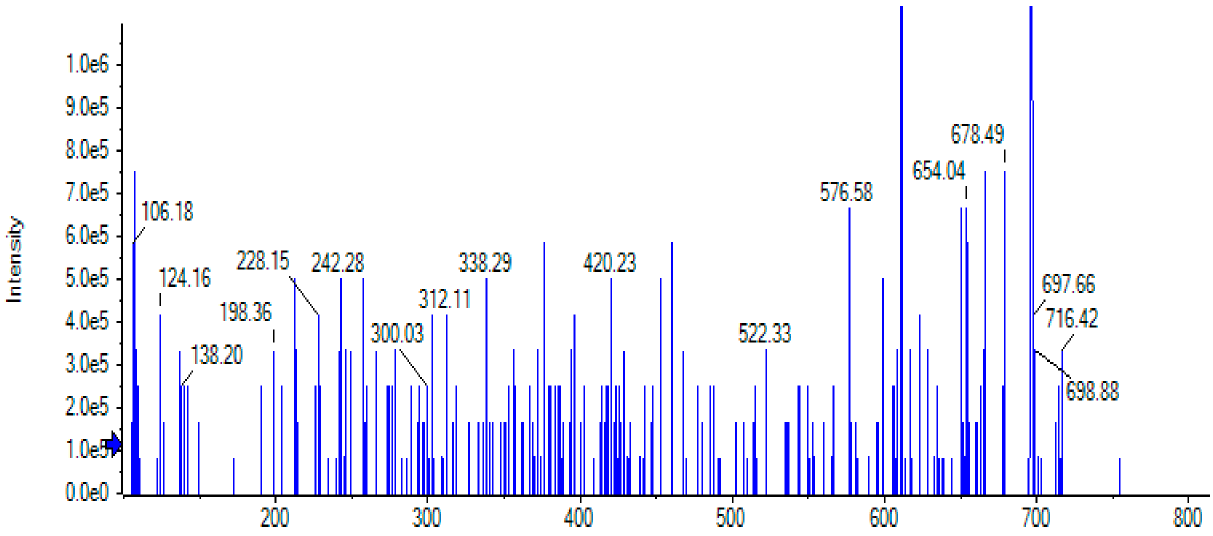

Figure S3b: The total ion chromatogram of 2D spot obtained from fermented buffalo milk with KGL4+WBS2A culture

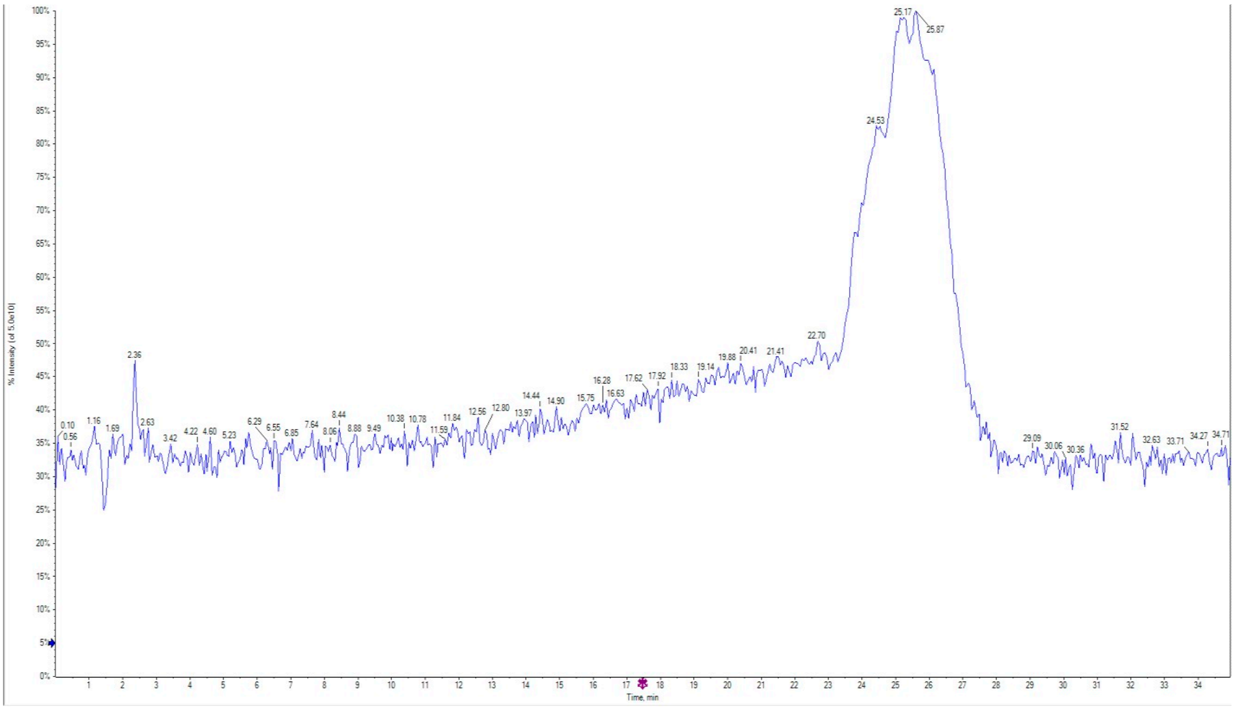

Figure S3c: The total ion chromatogram of 2D spot obtained from fermented camel milk with KGL4+WBS2A culture

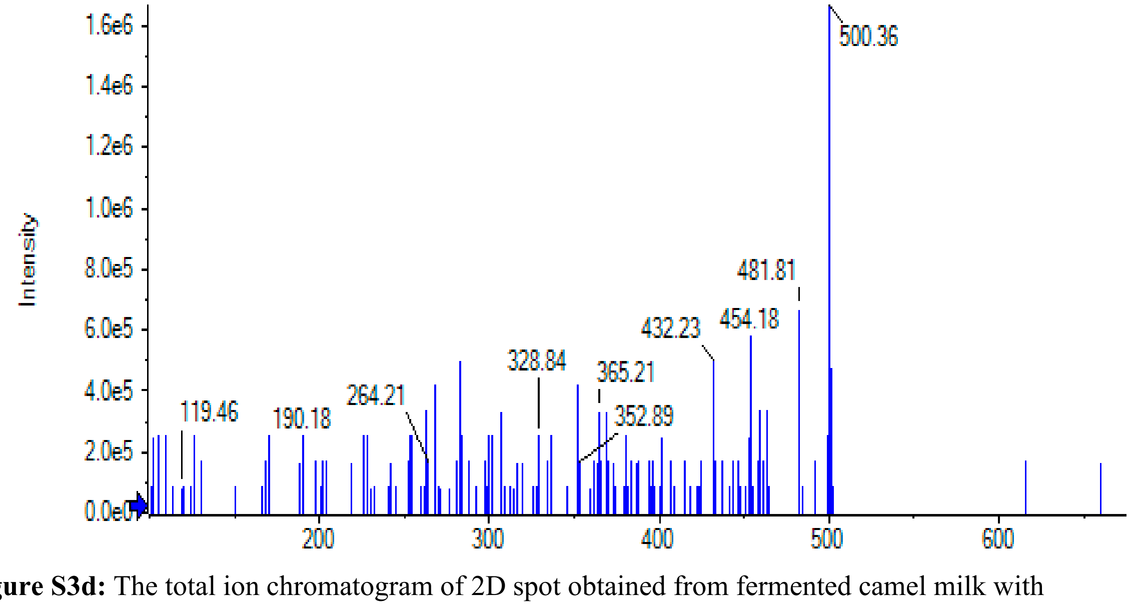

Figure S3d: The total ion chromatogram of 2D spot obtained from fermented camel milk with KGL4+WBS2A culture

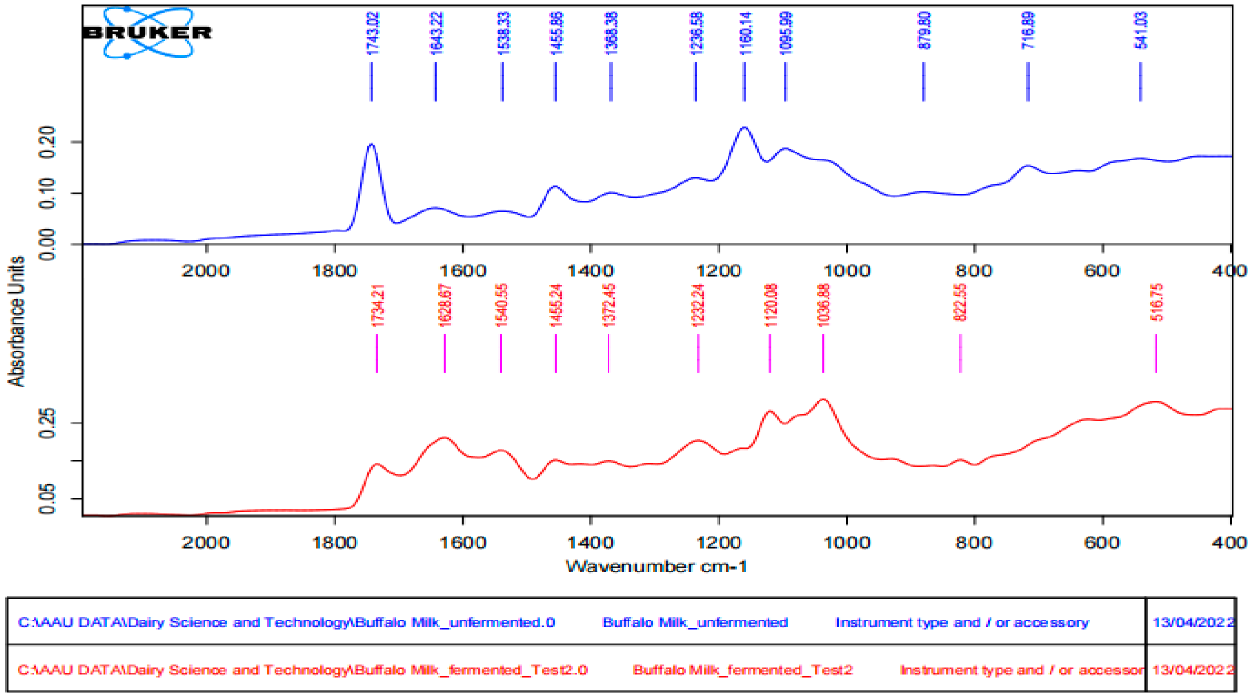

Figure S4a: Fourier Transform-Infrared Spectroscopy (FTIR) of buffalo milk fermented with KGL4+WBS2A

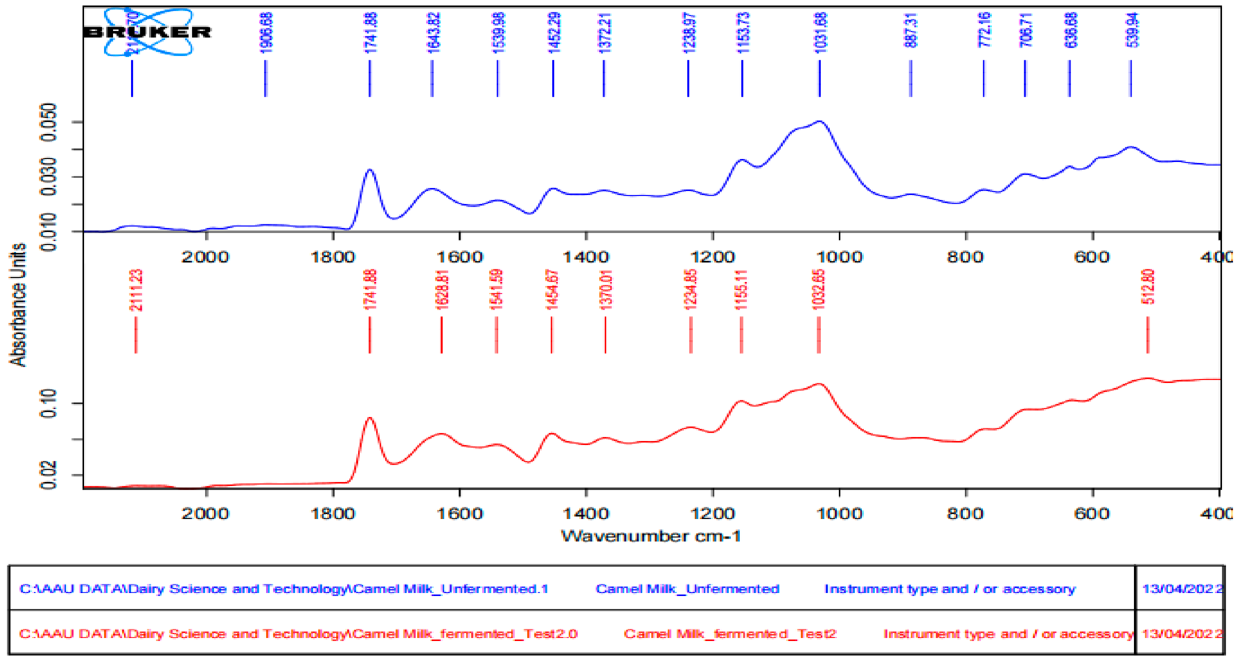

Figure S4b: Fourier Transform-Infrared Spectroscopy (FTIR) of camel milk fermented with KGL4+WBS2A

Table S1a. Amino acid sequence obtained from 2D-PAGE of fermented buffalo milk (KGL4+WBS2A) searched on AHTPBD database

Sequence	ID	PMID/ LINK	Matched Sequence	Molecular Weight	Source	Reference
SCQAQPT TMTR	2092	2092_link	GGR**PT**Y	649.7	Milk derived	[54]
	2235	2235_link	VRL**PT**	584.72	Milk	[54]
	1155	1155_link	KYPV**QP**FTESQSLTL	1737.97	Milk	[55]
	1166	1166_link	L**QP**	356.42	Milk	[56]
	1189	1189_link	TPVVVPPFL**QP**	1193.45	Milk	[56]
	2288	2288_link	I**QP**	356.42	Milk derived	[57]
	2560	24915368	S**QP**K	458.51	Goat milk hydrolysate	[58]
	2582	24915368	TPVVVPPFL**QP**	1193.45	Cheese whey protein	[58]
	2758	24915368	L**QP**	356.42	Cheese	[58]
EMPFPK	1132	1132_link	**EMPFPK**	747.91	Milk	[56]
	5528	5528_link	**EMPFPK**	747.91	Goat milk protein	[59]
	5903	5903_link	**EMPFPK**	734	Milk (bovine)	[60]
	6512	6512_link	**EMPFPK**	747.899	Bovine milk protein	[60]
	1204	1204_link	VY**PFP**G	678.79	Milk	[56]
	1662	1662_link	VY**PFP**G	678.79	Casein	[61]
	1771	1771_link	FVA**PFP**EV	905.06	Casein	[62]
TTMPLW	1191	1191_link	**TTMPLW**	747.91	Milk	[56]
	4143	22249830	**TTMPLW**	747.91	Milk proteins	[6]
	4275	21773582	**TTMPLW**	747.91	Bovine casein proteins	[63]
	5906	5906_link	**TTMPLW**	748	Milk (bovine)	[60]
	5835	5835_link	**TTMPLW**	748	Milk (bovine casein)	[64]
	1177	1177_link	**PLW**	414.5	Milk	[56]
	1191	1191_link	TTM**PLW**	747.91	Milk	[56]
	1658	1658_link	TTM**PLW**	747.91	Casein	[61]

	2245	2245_link	**PLW**	414.5	Milk derived	[57]
	4143	22249830	TTM**PLW**	747.91	Milk proteins	[6]
	5835	5835_link	TTM**PLW**	748	Milk (bovine casein)	[64]
HPHPHLSF MAIPPK	4298	4298_link	MAI**PPK**	655.85	Goat milk protein and cheeses	[65]
	4299	4299_link	MAIP**PKK**	784.03	Goat milk protein and cheeses	[65]
	4324	4324_link	MAI**PPK**	655.85	Sheep milk and cheeses proteins	[65]
	4325	4325_link	MAIP**PKK**	784.03	Sheep milk and cheeses proteins	[65]
	1335	1335_link	M**AIPP**KK	784.03	Casein	[66]
	4145	22249830	M**AIPP**KK	784.03	Milk proteins	[6]
	4298	4298_link	M**AIPP**K	655.85	Goat milk protein and cheese	[65]
	4299	4299_link	M**AIPP**KK	784.03	Goat milk protein and cheeses	[65]
	4324	4324_link	M**AIPP**K	655.85	Sheep milk and cheeses proteins	[65]
	4325	4325_link	M**AIPP**KK	784.03	Sheep milk and cheeses proteins	[65]
FFNDKIAK	1792	1792_link	FSDK**IAK**YIPIQ	1422.69	Casein	[62]
	4153	22249830	**IAK**	330.43	Milk proteins	[6]
	4322	4322_link	**IAK**	330.43	Sheep milk and cheeses proteins	[65]
	4142	22249830	**FF**VAPFPGVFGK	1312.58	Milk proteins	[6]
	4272	21773582	**FF**VAPFPEVFGK	1384.64	Bovine casein proteins	[63]
	4273	21773582	**FF**VAP	579.7	Bovine casein proteins	[63]
	5534	5534_link	**FF**VAPFPEVFGK	1384.64	Goat milk protein	[59]
	5834	5834_link	**FF**VAPFPEVFGK	1385	bovine casein	[64]
ALPMHIR	1122	1122_link	ALPMHIR	837.05	Milk	[56]
	1322	1322_link	**ALPMHIR**	837.05	Beta Lactoglobulin	[66]
	4266	21773582	**ALPMHIR**	837.05	Bovine whey proteins	[63]
	4328	4328_link	**ALPMHIR**	837.05	Sheep milk and cheeses proteins	[65]
	1122	1122_link	**ALPMH**IR	837.05	Milk	[56]
	1321	1321_link	**ALPMH**	567.7	Beta Lactoglobulin	[66]
	1322	1322_link	**ALPMH**IR	837.05	Beta Lactoglobulin	[66]

	5841	5841_link	F**ALP**QYLK	979	bovine casein	[64]
	5840	5840_link	F**ALP**QY	738	bovine casein	[64]
IPAVFK	1146	1146_link	**IPA**	299.37	Milk	[56]
	1300	1300_link	**IPA**	299.37	Whole whey protein	[66]
	2261	2261_link	**IPA**	299.37	Milk derived	[57]
	4160	22249830	**IPA**	299.37	Milk proteins	[6]
	2262	2262_link	**VFK**	392.5	Milk derived	[57]
LDQWLCEK	2215	2215_link	L**EK**TG	546.62	Milk	[54]
	2236	2236_link	VRP**EK**	627.74	Milk	[54]
	4295	4295_link	TDV**EK**	590.63	Goat milk protein and cheeses	[65]
	1197	1197_link	VGINY**WL**AHK	1200.41	Milk	[56]
	1205	1205_link	**WL**AHK	653.78	Milk	[56]
	1305	1305_link	**WL**AHK	653.78	Whole whey protein	[66]
	1331	1331_link	VGINY**WL**AHKHK	1465.72	Alpha-Lactoglobulin	[66]
AVPYPQR	1125	1125_link	**AVPYP**	545.64	Milk	[56]
	1126	1126_link	**AVPYP**QR	829.95	Milk	[56]
	1657	1657_link	**AVPYP**QR	829.95	Casein	[61]
	4144	22249830	**AVPYP**QR	829.95	Milk proteins	[6]
	1126	1126_link	AVPY**PQR**	829.95	Milk	[56]
	1178	1178_link	**PQR**	399.45	Milk	[56]
	1126	1126_link	**AVPYPQR**	829.95	Milk	[56]
	1657	1657_link	**AVPYPQR**	829.95	Casein	[61]
	4269	21773582	**AVPYPQR**	829.95	Bovine casein proteins	[63]

Table S1b Amino acid sequence obtained from 2D-PAGE of fermented camel milk (KGL4+WBS2A) searched on AHTPBD database

**Sequence**	**ID**	**PMID/LINK**	**Matched Sequence**	**Molecular Weight**	**Source**	**Reference**
TDVMPQ	1165	1165_link	L**PQ**NIPPLTQTPVVVPPFLQPEVMGVSK	3026.63	Milk	[56]

WW	1178	1178_link	**PQ**R	399.45	Milk	[56]
	1210	1210_link	YKV**PQ**L	746.9	Milk	[56]
	1343	1343_link	YKV**PQ**L	746.9	Casein	[66]
	1665	1665_link	YKV**PQ**L	746.9	Casein	[61]
	1769	1769_link	F**PQ**YLQY	958.08	Casein	[62]
	1787	1787_link	FP**PQ**SVL	786.93	Casein	[62]
	1165	1165_link	LPQNIPPLTQTPVVVPPFLQPE**VM**GVSK	3026.63	Milk	[56]
	1791	1791_link	QSWMHQPHQPLPPT**VM**	1914.23	Casein	[62]
EKTFLLYS CPHR	1798	1798_link	FVAPFPEVFGK**EK**VNE	1837.11	Casein	[62]
	2215	2215_link	L**EK**TG	546.62	Milk	[54]
	2236	2236_link	VRP**EK**	627.74	Milk	[54]
	1163	1163_link	**LLY**QQPVLGPVRGPFPIIV	2106.58	Milk	[56]
SSHPYLEQ LY	2257	2257_link	**SHP**	339.35	Milk derived	[57]
	1143	1143_link	**HP**FAQTQSLVYP	1387.56	Milk	[56]
	1145	1145_link	I**HP**FAQTQSLVYP	1500.72	Milk	[56]
	1790	1790_link	AR**HP**HPHLSF	1198.35	Casein	[62]
	2093	2093_link	I**HP**	365.43	Milk derived	[54]
	2257	2257_link	**SHP**	339.35	Milk derived	[57]
	2760	24915368	RPK**HP**IKHQ	1140.36	Gouda cheese	[58]
IDSGLYLG SNYITAIR	1163	1163_link	L**LY**QQPVLGPVRGPFPIIV	2106.58	Milk	[55]
	2223	2223_link	**LY**W	480.56	Milk	[54]
	1138	1138_link	**GL**DIQK	672.78	Milk	[56]
	1208	1208_link	Y**GL**F	498.58	Milk	[56]
	1296	1296_link	**GL**DIQK	672.78	Whole whey protein	[66]
	1328	1328_link	Y**GL**	351.4	Alpha-Lactoglobulin	[66]
	1329	1329_link	Y**GL**F	498.58	Alpha-Lactoglobulin	[66]
	2682	24915368	**GLY**	351.4	Royal jelly	[58]
FDEFLSQS	1160	1160_link	LDAQS**AP**LR	970.09	Milk	[56]

CAPGSDPR	1195	1195_link	VAP	285.34	Milk	[56]
	1299	1299_link	DAQS**AP**LRVY	1119.24	Whole whey protein	[66]
	1311	1311_link	LDAQS**AP**LR	970.09	Beta Lactoglobulin	[66]
	1312	1312_link	S**AP**LRVY	804.94	Beta Lactoglobulin	[66]
	1798	1798_link	FV**AP**FPEVFGKEKVNE	1837.11	Casein	[62]
	1204	1204_link	VYPF**PG**	678.79	Milk	[56]
	1662	1662_link	VYPF**PG**	678.79	Casein	[61]
	1779	1779_link	LNV**PG**E	627.7	Casein	[62]
	2577	24915368	VYPF**PG**	678.79	Cheese whey protein	[58]
	2762	24915368	YPF**PG**PIPN	1001.15	Gouda cheese	[58]

Table S1c. Amino acid sequence obtained from 2D-PAGE of fermented buffalo milk (KGL4+WBS2A) searched on BIOPEP database

**Sequence**	**ID**	**Matched Sequence**	**Molecular Mass**	**Source**	**Reference**
SCQAQPTTMTR	8632	FHTSGYDT**QA**	1126.1300	**α-Lactalbumin**	[67]
	8633	TSGYDT**QA**IV	1054.1040	**α-Lactalbumin**	[67]
	8634	DT**QA**IVQNND	1117.1250	**α-Lactalbumin**	[67]
	8649	VGGSDL**QA**LK	987.1100	macroalga Palmaria palmata	[68]
	8735	LSERRMLLRKEK**QA**Q	1886.2430	Common Bean	[69]
EMPFPK	8747	YINQ**MP**QKSRE	1393.5690	egg-yolk protein	[70]
	8750	YINQ**MP**QKSREA	1464.6480	egg-yolk protein	[70]
	8740	YINQ**MP**QKSREA	1464.6480	egg-yolk protein	[70]
	9408	**FP**GPIPD	741.8290	**gouda cheese**	[71]
	9410	YP**FP**GPIPD	1002.1160	**gouda cheese**	[71]
TTMPLW	8747	YINQ**MP**QKSRE	1393.5690	egg-yolk protein	[70]
	8750	YINQ**MP**QKSREA	1464.6480	egg-yolk protein	[70]
	8739	YINQ**MP**QKSRE	1393.5690	egg-yolk protein	[70]

	10214	V**PL**VM	557.7410	broccoli	[72]
	9848	LL**PL**PVLK	892.1810	soy protein	[73]
	9703	L**PL**LR	610.7850	walnut (Juglans mandshurica Maxim)	[73]
	9705	L**PL**LR	610.7850	walnut (Juglans mandshurica Maxim)	[73]
	9692	L**PL**LR	610.7850	walnut (Juglans mandshurica Maxim)	[73]
	9547	Y**PL**	391.4470	sardine muscle hydrolyzate	[74]
	9388	YY**PL**	554.6170	sardine muscle hydrolyzate	[74]
HPHPHLSF MAIPPK	9409	V**PP**FIQPE	926.0680	**gouda cheese**	[71]
	10115	YPFPGP**IP**N	1001.1310	**gouda cheese**	[71]
FFNDKIAK	8644	A**IA**V	372.4510	macroalga Palmaria palmata	[68]
	8645	L**IA**P	412.5160	macroalga Palmaria palmata	[68]
	8646	I**IA**P	412.5160	macroalga Palmaria palmata	[68]
	8627	IPAVF**KI**DAL	1086.3280	**α-Lactalbumin**	[67]
	8552	R**ND**DLNYIQ	1150.1950	egg-yolk protein	[70]
	8634	DTQAIVQN**ND**	1117.1250	**α-Lactalbumin**	[67]
ALPMHIR	9848	LLP**LP**VLK	892.1810	soy protein	[73]
	9692	**LP**LLR	610.7850	walnut (Juglans mandshurica Maxim)	[73]
	9703	**LP**LLR	610.7850	walnut (Juglans mandshurica Maxim)	[73]
	9705	**LP**LLR	610.7850	walnut (Juglans mandshurica Maxim)	[73]
	9198	FFRSKLLSDGAAAAKGAL**LP**QYW	2510.9090	cumin seeds	[75]
	9200	RCMAFLLSDGAAAAQQL**LP**QYW	2470.7120	cumin seeds	[75]
	8619	TPEVDDE**AL**EK	1245.2940	**whey protein concentrate**	
	8624	LAHK**AL**CSEK	1116.1640	**α-Lactalbumin**	[67]
IPAVFK	8620	**IPAVF**	545.6660	**whey protein concentrate**	[76]
	8621	**IPAVF**K	673.8400	**whey protein concentrate**	[76]
	8627	**IPAVF**KIDAL	1086.3280	**α-Lactalbumin**	[67]

	8621	**IPAVFK**	673.8400	**whey protein concentrate**	[76]
	8627	**IPAVFK**IDAL [97]	1086.3280	**α-Lactalbumin**	[67]
LDQWLCE K	8619	TPEVDDEAL**EK**	1245.2940	**whey protein concentrate**	[76]
	8624	LAHKALCS**EK**	1116.1640	**α-Lactalbumin**	[67]
	8626	LCS**EK**LDQWL	1251.2830	**α-Lactalbumin**	[67]
	8625	WCKD**DQ**NPHS	1246.1390	**α-Lactalbumin**	[67]
	8626	LCSEKL**DQ**WL	1251.2830	**α-Lactalbumin**	[67]
	8635	CKD**DQ**NPHSS	1147.0060	**α-Lactalbumin**	[67]
AVPYPQR	10213	**VP**LVM	557.7410	broccoli	[72]
	10214	**VP**LVM	557.7410	broccoli	[72]

Table S1d. Amino acid sequence obtained from 2D-PAGE of fermented camel milk (KGL4+WBS2A) searched on BIOPEP database

**Sequence**	**ID**	**Matched Sequence**	**MolecularMass**	**Source**	**Reference**
TDVMPQWW	8739	YINQ**MP**QKSRE	1393.5690	egg yolk protein hydrolysate	[70]
	8740	YINQ**MP**QKSREA	1464.6480	egg yolk protein hydrolysate	[70]
	8747	YINQ**MP**QKSRE	1393.5690	egg yolk protein hydrolysate	[70]
	8750	YINQ**MP**QKSREA	1464.6480	egg yolk protein hydrolysate	[70]
	8626	LCSEKLD**QW**L	1251.2830	**α-Lactalbumin**	[67]
EKTFLLYSCP HR	9692	LP**LL**R	610.7850	walnut (Juglans mandshurica Maxim)	[73]
	9703	LP**LL**R	610.7850	walnut (Juglans mandshurica Maxim)	[73]
	9705	LP**LL**R	610.7850	walnut	[73]
	9198	FFRSK**LL**SDGAAAAKGALLPQYW	2510.9090	cumin seeds	[75]
	9200	RCMAF**LL**SDGAAAAQQLLPQYW	2470.7120	cumin seeds	[75]
	10110	YL**YS**PAY	875.9460	broccoli	[78]
SSHPYLEQLY	8625	WCKDDQN**PH**S	1246.1390	**α-Lactalbumin**	[67]
	8635	CKDDQN**PH**SS	1147.0060	**α-Lactalbumin**	[67]
	8636	QN**PH**SSNICN	1130.0220	**α-Lactalbumin**	[67]

	8720	RE**YL**LVAQ	991.1370	Common Bean (Phaseolus vulgaris)	[69]
	10110	**YL**YSPAY	875.9460	broccoli	[77]
IDSGLYLGSN YITAIR	10102	SQHIS**TA**GMEASGTSNMKF	1984.1920	Changii radix hydrolysates	[78]
	9202	DPAQPNYPW**TA**VLVFRH	2011.2540	cumin seeds	[75]
	8720	RE**YL**LVAQ	991.1370	Common Bean (Phaseolus vulgaris)	[69]
	10110	**YL**YSPAY	875.9460	broccoli	[77]
FDEFLSQSCA PGSDPR	8500	**APG**	243.2480	**bovine whey proteins**	[67]
	9549	Y**PG**	335.3390	sardine muscle hydrolyzate	[74]
	9408	F**PG**PIPD	741.8290	**gouda cheese**	[71]
	9410	YPF**PG**PIPD	1002.1160	**gouda cheese**	[71]

Table S2. Details of intermolecular forces arose as a result of interaction between selected peptides and target proteins used in this study

**Intermolecular interactions between human pancreatic alpha amylase (3BAI) and selected peptides**					
**FFNDKIAK**			**EKTFLLYSCPHR**		
**Peptide-protein residue pair**	**Interaction type**	**Bond distance (Å)**	**Peptide-protein residue pair**	**Interaction type**	**Bond distance (Å)**
LYS5-ASP300	Salt bridge	1.58	ARG12-ASP147	Salt bridge	1.66
PHE1-GLU240	Charge-charge	5.01	GLU1-LYS261	Charge- charge,	5.08
LYS5-ASP197	Charge-charge	5.48	THR3-GLY308	Hydrogen bond	1.88
LYS8-ASP356	Charge-charge	4.05	CYS9-THR163	Hydrogen bond	1.98
LYS5-THR163	Hydrogen bond	1.71	HIS11-ALA106	Hydrogen bond	1.87
ASN3-ASP300	Hydrogen bond	1.88	ARG12-ASP147	Carbon-hydrogen bond	3.42
ASP4-HIS305	Hydrogen bond	1.80	PHE4-GLU240	Pi-Anion	3.75
LYS8-HIS305	Pi-Cation	4.79	LYS2-LEU237	Alkyl	4.22
ASP4-HIS305	Pi-Anion	3.39	CYS9-LEU165	Alkyl	5.01
PHE2-ILE235	Pi-Sigma	3.83	PRO10-TRP59	Pi-Alkyl	4.96
PHE2-TYR151	Pi-Pi Stacked	5.57	HIS11-ALA106	Pi-Alkyl	4.26
ILE6-LEU162	Alkyl	4.45			
LYS5-HIS305	Pi- Alkyl	5.01			
PHE2-LYS200	Pi- Alkyl	4.80			
**Intermolecular interactions between human maltase-glucoamylase (3CTT) and selected peptides**					
**FFNDKIAK**			**EKTFLLYSCPHR**		
**Peptide-protein residue pair**	**Interaction type**	**Bond distance (Å)**	**Peptide-protein residue pair**	**Interaction type**	**Bond distance (Å)**
ASP4-ARG334	Salt bridge	2.60	ARG12 -ASP542	Salt bridge	1.68
ASP4-ARG334	Salt bridge	2.86	ARG12-ASP443	Salt bridges	1.91
LYS5-ASP542	Salt bridge	1.82	ARG12:-ASP203	Charge-charge	4.68

LYS5-ASP443	Salt bridge	1.70	ARG12-ASP443	Charge-charge	2.78
ASP4-ARG334	Charge-charge	5.55	ARG12-ASP542	Charge-charge	4.76
PHE2-TYR605	Hydrogen bond	2.43	TYR299-ARG12	Hydrogen bonds	2.43
PHE2-THR205	Pi-Sigma	3.97	HIS11-TYR605	Hydrogen bond	1.93
LYS5-TYR209	Pi-alkyl	4.56	ARG12-ASP542	Hydrogen bond	1.63
LYS5-TRP406	Pi-alkyl	5.29	ARG12-MET444	Hydrogen bond	2.15
LYS8-PHE450	Pi-alkyl	5.49	LYS480-TYR7	Carbon-hydrogen bond	3.42
LYS5-PHE575	Pi-alkyl	5.22	ARG12-ASP203	Carbon-hydrogen bond	3.33
PHE2-ALA576	Pi-alkyl	5.29	MET444-ARG12	Sulfur-X	3.07
